# Genes involved in floral meristem in tomato exhibit drastically reduced genetic diversity and signature of selection

**DOI:** 10.1186/s12870-014-0279-2

**Published:** 2014-10-19

**Authors:** Guillaume Bauchet, Stéphane Munos, Christopher Sauvage, Julien Bonnet, Laurent Grivet, Mathilde Causse

**Affiliations:** INRA, UR1052, Génétique et Amélioration des Fruits et Légumes (GAFL), 67 Allée des Chênes Domaine Saint Maurice – CS60094, 84143 Montfavet Cedex, France; Syngenta Seeds, 12 chemin de l’Hobit, 31790 Saint Sauveur, France; Present address: INRA, UMR CNRS-INRA 441-2594, 24 Chemin de Borde Rouge – Auzeville - CS 52627, 31326 Castanet Tolosan Cedex, France

**Keywords:** Tomato, *S. lycopersicum*, Meristem, Signature of selection

## Abstract

**Background:**

Domestication and selection of crops have notably reshaped fruit morphology. With its large phenotypic diversity, tomato (*Solanum lycopersicum*) illustrates this evolutive trend. Genes involved in flower meristem development are known to regulate also fruit morphology. To decipher the genetic variation underlying tomato fruit morphology, we assessed the nucleotide diversity and selection footprints of candidate genes involved in flower and fruit development and performed genome-wide association studies.

**Results:**

Thirty candidate genes were selected according to their similarity with genes involved in meristem development or their known causal function in *Arabidopsis thaliana*. In tomato, these genes and flanking regions were sequenced in a core collection of 96 accessions (including cultivated, cherry-type and wild relative accessions) maximizing the molecular diversity, using the Roche 454 technology. A total amount of 17 Mb was sequenced allowing the discovery of 6,106 single nucleotide polymorphisms (SNPs). The annotation of the 30 gene regions identified 231 exons carrying 517 SNPs. Subsequently, the nucleotide diversity (*π*) and the neutral evolution of each region were compared against genome-wide values within the collection, using a SNP array carrying 7,667 SNPs mainly distributed in coding sequences.

About half of the genes revealed footprints of selection and polymorphisms putatively involved in fruit size variation by showing negative Tajima’s D and nucleotide diversity reduction in cultivated tomato compared to its wild relative. Among the candidates, *FW2.2* and *BAM1* sequences revealed selection footprints within their promoter regions suggesting their potential involvement in their regulation. Two associations co-localized with previously identified loci: *LC* (locule number) and *Ovate* (fruit shape).

**Conclusion:**

Compared to whole genome genotypic data, a drastic reduction of nucleotide diversity was shown for several candidate genes. Strong selection patterns were identified in 15 candidates highlighting the critical role of meristem maintenance genes as well as the impact of domestication on candidates. The study highlighted a set of polymorphisms putatively important in the evolution of these genes.

**Electronic supplementary material:**

The online version of this article (doi:10.1186/s12870-014-0279-2) contains supplementary material, which is available to authorized users.

## Background

Understanding the evolutionary basis of plant variation can be reached through the identification of the molecular mechanisms responsible for the large diversity in plant architecture [1,2]. Evolutionary changes in fruit shape and size has played a key role in the morphological diversification of plant species [3]. Meristem regulation growth is hypothesized to play a major role in sculpting the plant and fruit morphology [4,5]. Its developmental regulation occurs at several levels, including (i) meristem maintenance, (ii) floral organ identity and (iii) floral meristem identity [6-8]. Ovary size partly explained fruit weight, which is first regulated in the meristem [9]. Floral meristem size may impact cell number that will form carpel primordium and subsequent number [10,11]. We hypothesized that variation in genes controlling meristem development and expressed very early in flower/fruit development could be good candidates for fruit size variation.

*Arabidopsis thaliana* is the standard reference for plant biology [12] and a premier model system for molecular and genetic analyses of meristem development [13]. However, the tomato fruit model system proposed by Gillaspy has shown its importance to decipher early developmental determinants, cell cycle steps and organ number determination [14-16]. Together with its ease to cultivate, short life cycle, rich genetic resources, relatively small genome size, available reference genome sequence [17], tomato (*Solanum lycopersicum*) has become a reference in fruit development studies and opens perspectives for a wider understanding of domestication process in fleshy fruit species [18]. Major QTLs involved in the evolution of fruit size and shape have been identified and a few underlying genes cloned [19,20]. For example, the fruit weight QTL *FW2.2* encodes a negative regulator of cell proliferation [21]. Regarding fruit shape, *OVATE* encodes an hydrophilic protein where a single mutation induces a stop codon causing a transition from round to pear shaped tomato fruit [22]. Moreover, two loci, *FASCIATED* and *LC,* that have pleiotropic effects on fruit shape and size [23], determine the locule number: *FASCIATED* encodes a YABBY like transcription factor [24] and *lc* mutation is close to the *WUSCHEL* gene involved in meristem maintenance [25]. Other genes are known [24] or hypothesized to be linked to meristem development [26], but a large genetic potential remains to be revealed [27].

Population genetic studies offer a powerful way to evaluate the molecular evolution of biological mechanisms and to assess the contribution of selection in shaping crop genetic variation and identify related constrains [28,29]. Recently, the Genome Wide Association (GWA) strategy that takes the advantage of natural populations and their increased recombination events [30] has been proposed to decipher the genetic architecture of traits linked to domestication [31]. GWA relies on linkage disequilibrium (LD) - non-random association of alleles [32] - and thus on recombination which occurred during meiosis events. In tomato, most recent GWA studies related to fruit size and shape were limited to a single chromosome [33], used a low density marker set [34] or a limited number of agronomical traits collected from public databases [35]. A complementary approach is to compare diversity patterns across species and look for signature of selection over the genome [36].

Here, we describe the patterns of sequence variation of 30 candidate genes in a tomato core collection composed of 96 accessions. The accessions were selected to represent the maximum diversity of a large tomato panel previously described [37]. The set was composed of 17 *S. lycopersicum* (SL) (including Heinz1706, the reference sequenced genome), 63 *S.l. cerasiforme* (SLC), 12 *S. pimpinellifolium* (SP), and four other wild species (WT). Candidate genes were selected for their known function related to tomato fruit size and shape and/or for their involvement in meristem development and maintenance. Using the sequence dataset obtained for the 30 large amplicons covering the genes, nucleotide diversity and signatures of selection were explored. We estimated a set of population genetic parameters (i.e. d_N_/d_S_, Tajima’s D) to evidence non-neutral processes operating on meristem regulation. We compared these values with those assessed at the whole genome level using a SNP array. Several genes under a strong reduction of diversity in cultivated tomato were identified. Associations with locule number and fruit shape were detected.

## Results

### Candidate gene selection

**Figure 1:**
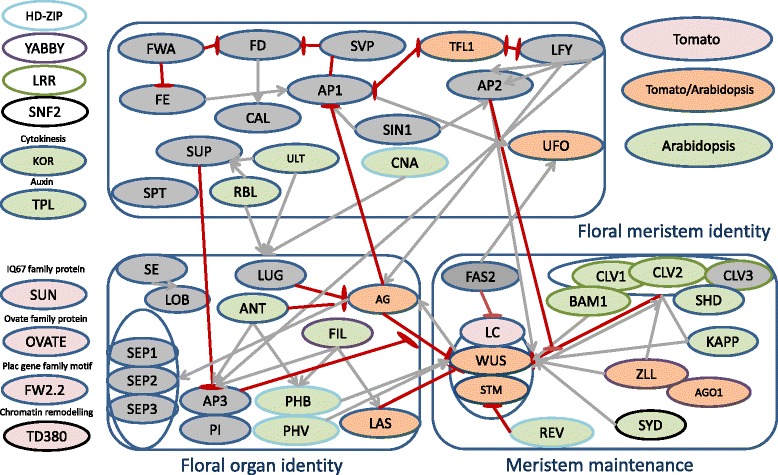
**A composite view of 50 genes involved in meristem development and their main pathways aggregated from literature review.** Genes characterized in *A. thaliana* are shown with a green background. Genes with known orthologs in *A. thaliana* and *S. lycopersicum* are in orange background. Genes initially characterized in *S. lycopersicum* are in a red background. Genes not involved in this study are shown with a grey background. Red arrows suggest a negative feedback between two gene entities. Grey arrows suggest activation. Colored circle highlight multiple genes from the same family (HD-ZIP, YABBY, LRR and SNF2). For candidate genes references, see Table 2.

We first selected 50 candidate genes from the literature. Figure 1 illustrates their classification according to their function and the known interactions between candidates. Among them, 30 genes were retained according to their specificity and success of PCR amplification on the 96 accessions. The 30 candidates included 12 genes involved in meristem maintenance, 6 in floral organ identity and 5 in floral meristem identity. Six other candidates were previously characterized as involved in tomato fruit morphology and two non-coding sequences (one covering the polymorphisms responsible for the *LC* QTL and a non coding region randomly selected, further named as *“Non Coding”*) were also included. Interestingly, we could not identify any ortholog of CLV3 in tomato. Six genes adjacent to the candidates were also partially covered by long range PCR. They were included in the study as they are closely linked to the candidates. Table 1 lists the candidate genes studied as well as their genomic positions in tomato genome and ortholog ontology in *Arabidopsis thaliana*. Their genomic positions on the reference genome (v2.40) are provided in Additional file 1.

**Table 1:** **Annotation of the candidate genes**

**Targeted tomato candidate genes and related annotation**							
**Gene**	**Gene abbreviation**	**Arabidopsis gene id**	**Tomato gene id**	**Tomato chromosome**	**Pathway compartment**	**Arabidopsis annotation**	**Gene_references**
TOPLESS	TPL	AT1G15750.1	Solyc03g117360.2.1	SL2.40ch03	Auxin pathway	WD-40 repeat protein-like	[38]
KORRIGAN 1	KOR1	AT5G49720.1	Solyc01g102580.2.1	SL2.40ch01	Cytokinesis	Endo 1,4 b glucanase	[39-41]
CORONA	CNA	AT1G52150.1	Solyc03g120910.2.1	SL2.40ch03	Floral meristem identity	HD ZIP III protein	[42-44]
ULTRAPETALA1	ULT1	AT4G28190.1	Solyc07g054450.2.1	SL2.40ch07	Floral meristem identity	SAND-domain transcription factor	[45]
ARGONAUTE 1	AGO1	AT1G48410.1	Solyc03g098280.2.1	SL2.40ch03	Floral meristem identity	YABBY/AGO	[46-48]
REBELOTE	RBL	AT3G55510.1	Solyc02g081680.2.1	SL2.40ch02	Floral meristem identity	unknown function	[49]
UNUSUAL FLORAL ORGAN	UFO	AT1G30950.1	Solyc02g081670.1.1	SL2.40ch02	Floral meristem identity	F box protein	[50-53]
SELF PRUNING	SP/TFL1	AT5G03840.1	Solyc06g074350.2.1	SL2.40ch06	Floral meristem identity	PEBP protein	[54-56]
FILAMENTOUS FLOWER	FIL-YAB1	AT2G45190.1	Solyc01g091010.2.1	SL2.40ch01	Floral organ identity	YABBY	[57-59]
LATERAL SUPPRESSOR	LAS	AT1G55580.1	Solyc07g066250.1.1	SL2.40ch07	Floral organ identity	GRAS family transcription factor	[60-62]
PHABULOSA	PHB	AT2G34710.1	Solyc02g024070.2.1	SL2.40ch02	Floral organ identity	HD ZIP III	[42,63,64]
PHAVOLUTA	PHV	AT1G30490.1	Solyc08g066500.2.1	SL2.40ch08	Floral organ identity	HD ZIP III	[42,63,64]
AINTEGUMENTA	ANT	AT4G37750.1	Solyc04g077490.2.1	SL2.40ch04	Floral organ identity	AP2/ERF transcription factor family	[65-67]
AGAMOUS	AG	AT4G18960	Solyc02g071730.2.1	SL2.40ch02	Floral organ identity	MADS Box transcription factor	[68-71]
CLAVATA 1	CLV1	AT1G75820.1	Solyc04g081590.2.1	SL2.40ch04	Meristem maintenance	LRR Receptor kinase	[72,73]
KINASE ASSOCIATED PROTEIN PHOSPHATASE	KAPP	AT5G19280.1	Solyc01g079720.2.1	SL2.40ch01	Meristem maintenance	kinase-associated protein phosphatase	[74,75]
SHEPERD	SHD	AT4G24190.1	Solyc04g081570.2.1	SL2.40ch04	Meristem maintenance	ER-resident HSP90-like protein	[76]
SHOOTMERISTEMLESS	STM	AT1G62360.1	Solyc02g081120.2.1	SL2.40ch02	Meristem maintenance	KNOX1 homeobox protein	[77-79]
SPLAYED	SYD	AT2G28290.1	Solyc02g068560.2.1	SL2.40ch02	Meristem maintenance	SNF2chromatin remodelling protein	[80]
WUSCHEL	WUS	AT2G17950.1	Solyc02g083950.2.1	SL2.40ch02	Meristem maintenance	WOX family protein	[81-84]
ZWILLE/ARGONAUTE10	ZLL-PNH-AGO10	AT5G43810.1	Solyc09g082830.2.1	SL2.40ch09	Meristem maintenance	YABBY/AGO	[46,85]
CLAVATA 2	CLV2	AT1G65380.1	Solyc04g056640.1.1	SL2.40ch04	Meristem maintenance	LRR Receptor like protein	[86,87]
REVOLUTA	REV	AT5G60690.1	Solyc11g069470.1.1	SL2.40ch11	Meristem maintenance	HD ZIP III	[42,64]
BARELY ANY MERISTEM 1	BAM1	AT5G65700.1	Solyc02g091840.2.1	SL2.40ch02	Meristem maintenance	LRR-RLKs kinase	[88,89]
LOCULE NUMBER	LC	AT5G66240.2	Solyc02g083940.2.1	SL2.40ch02	NA	non coding region	[23,25,26,90]
FRUIT WEIGHT 2.2	FW2.2/ATPCR2	AT1G14870.1	Solyc02g090730.2.1	SL2.40ch02	NA	Plac gene family motif	[21,91-95]
OVATE*	OVATE/ATOFP7	AT2G18500.1	Solyc02g085500.2.1	SL2.40ch02	NA	Ovate family Protein	[22,58,96-98]
SUN*	SUN	AT5G03960.1	Solyc10g079240.1.1	SL2.40ch10	NA	IQ67 family protein	[99,100]
TD380*	TD380 /DDM1	AT5G66750.1	Solyc02g085390.2.1	SL2.40ch02	NA	SNF2 chromatin remodelling protein	[33,101]
*NON CODING*	NCD	non coding	non coding	SL2.40ch02	NA	non coding region	NA
**Gene**	**Gene abbreviation**	**Arabidopsis gene id**	**Tomato solyc id**	**Tomato chromosome**		**Arabidopsis annotation**	**Gene_references**
AHRD V1 ***- Q9LIL2_ARATH	EIF3C	AT3G56150.2	Solyc01g102570.2.1	SL2.40ch01	NA	eukaryotic translation initiation factor 3C	[102]
AHRD V1 *-*- MDTK	ZF14	AT1G58340.1	Solyc02g090740.2.1	SL2.40ch02	NA	MATE efflux family protein	[103]
Ovate protein	ATOFP6, OFP6	AT3G52525.1	Solyc02g085510.1.1	SL2.40ch02	NA	ovate family protein … 55 7e-09	[98]
Adenylosuccinate synthetase	ADSS	AT3G57610.1	Solyc02g085520.2.1	SL2.40ch02	NA	adenylosuccinate synthase	[104]
Glucosyltransferase	UGT71B8	AT3G21800.1	Solyc02g081690.1.1	SL2.40ch02	NA	UDP-glucosyl transferase 71B8	[105]
ATP dependent RNA helicase	DEA(D/H) Box	AT4G16630.1	Solyc04g081580.2.1	SL2.40ch04	NA	DEA(D/H)-box RNA helicase family protein	[106]
Os01g0786800 protein	Exporter	AT2G25737.1	Solyc02g085400.2.1	SL2.40ch02	NA	Sulfite exporter TauE/SafE family protein	[107]

Gene function, *Arabidopsis thaliana* and tomato gene ID, Tomato chromosome location (v2.40) and bibliographic references. Supplementary genes partially sequenced.

*Cloned tomato genes.

### Sequencing results

About a million of ≈ 350 bp reads were generated, while 852,500 reads were aligned onto 174,612 kb of the reference Heinz 1706 sequence (92.5% covered). Roche 454 sequencing process is known to induce a large amount of false INDELs, particularly in homopolymeric regions [108]. For the subsequent analysis, we thus only focused on SNP. Average read depth was 17X while the mapping percentage varied according to taxa from 93.7% in SL to 61.6% for the wild relative *S. pennellii*. Mapping on the reference genome success rate reached 92.7% of the reads. This proportion fell to 61.6%, 76.2%, 83.5%, 86.3% for the four wild accessions of *S. pennellii, S. habrochaites, S. chmielewskii, and S. pimpinellifolium* accessions, respectively. Interestingly *S. cheesmaniae* showed a high mapping rate (94.2%). This result indicates the limit of the alignment procedure for distant accessions. We increased mapping accuracy by *de novo* assembly and aligned 93.5%, 92.7%, 92.2% and 91.9% of WT, respectively, confirming the need to modify the procedure for the wild accessions.

### Sequence annotation

All the amplicons were annotated using ITAG 2.3 and classified as coding regions except the fragment “NON CODING”. After Open Reading Frame checking, the exon proportion per fragment ranged from 1% (*LC*) to 44% (*CLV2*). Sequence fragments covered 36 predicted gene entities (30 selected and 6 adjacent genes). Exon number (231) and their average size (170 bp) per candidate gene varied also significantly from 17 bp (*AG*) to 2,600 bp (*BAM1*) and exon number from 1 (*CLV2*, *LAS*) to 25 (*TPL*). Altogether, candidate genes and their flanking unigenes represented 40 kb of coding sequence or 27.8% of the targeted genomic sequence.

### Polymorphism discovery

**Figure 2:**
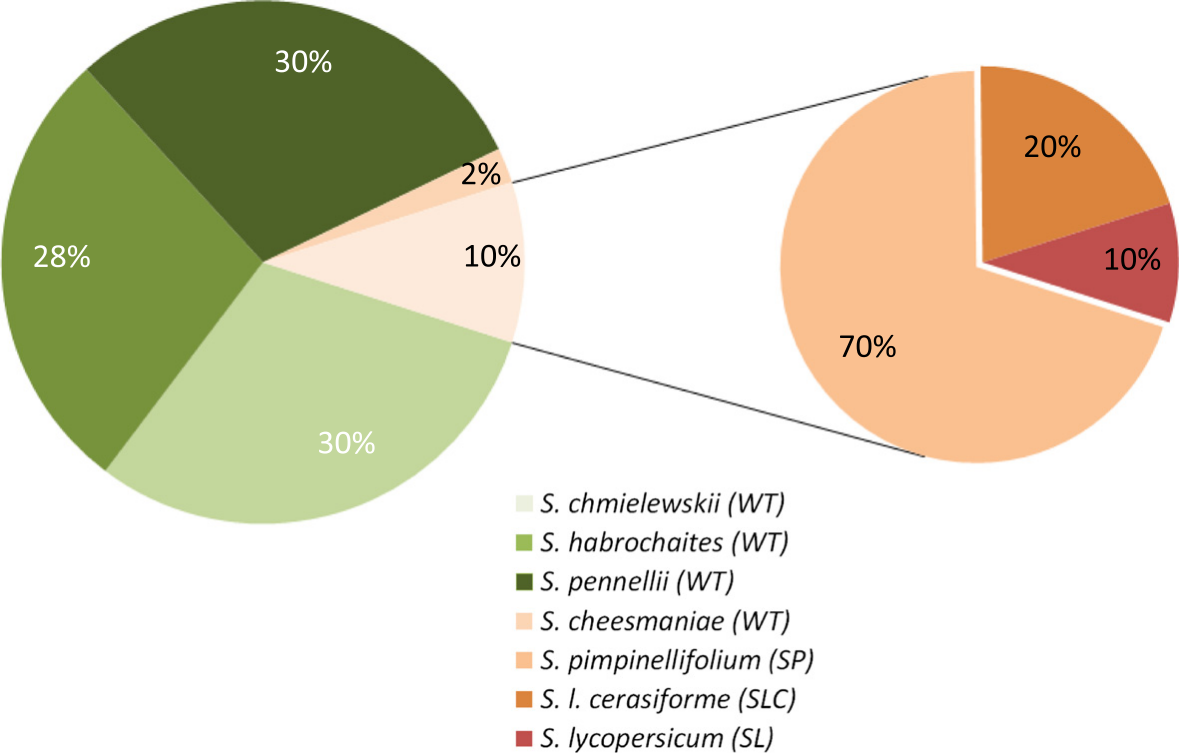
**SNP distribution among taxa.** The percentages correspond to taxon specific SNP. In green: green mature fruit species (*S.chmielewski*i, *S.habrochaites*, *S.pennellii*). In orange: orange (*S. cheesmanii*) or red mature fruit species (*S. pimpinellifolium*, *S.L. cerasiforme* and *S.lycopersicum*).

We detected 3,747 unique SNPs in the three main groups (SL, SLC and SP) and 2,359 SNP by *de novo* assembly in the wild taxa, for a total of 6,106 SNPs. The average SNP density by taxa and accession reached 1 SNP every 2,889 bp for SL, 1 SNP every 1,401 bp for SLC and 1 SNP every 406 bp for SP. Within the wild accessions, *S. cheesmaniae* showed the lowest diversity (1 SNP every 1,297 bp). Other wild accessions reached 1 SNP every 96 bp on average (Figure 2). SNP distribution in terms of coding/noncoding region is detailed in Table 2. Regarding SNP identified within the coding sequences (CDS), 423 of these were identified when mapped on the reference (1 SNP every 1,126 bp) and 134 by de *novo* assembly (1 SNP every 2,169 bp). The most polymorphic locus was *UFO* (1 SNP every 32 bp). The least polymorphic loci were LC (1 SNP every 8,807 bp) followed by *AG* (1 SNP every 5,074 bp). We also genotyped the SL, SLC, and SL in the collection (referred further as the 92 accessions) with the SolCAP SNP array,7,667 SNPs [109]. Tp perform GWAS, we filtered for rare alleles and missing data and obtained a5,795 SNPs set. As a cross validation between sequencing and genotyping data, 22 SNP markers of the SolCAP array overlapped the re-sequenced regions. All of them were also identified using the 454 sequencing results. Over the 6,106 SNPs, SnpEff tool identified in the target genes, 432 intragenic (=within CDS) polymorphisms (7%), 568 intergenic (9%), 284 synonymous (4.6%) and 120 corresponded to non-synonymous mutations (2%). More specifically, one synonymous stop (*CNA*), two splice donors (*ZLL*; *REV*), one stop lost (*OVATE*) and one stop gained (*SUN*) were identified. Nine candidate genes (*AG, CLV1*, *PHV*, *WUS*, *LC*, *KOR1*, *RBL*, *ANT* and *TD380*) did not show any non-synonymous mutation.

**Table 2:** **Effects of the SNP detected in coding sequences in the collection of 92 red fruited accessions**

**Gene name**	**Gene ID**	**exon number**	**CDS lenght**	**exon length sequenced**	**SNP in coding sequences**	**Non synonymous SNPs**	**Synonymous SNPs**	**SNP in 3’ UTR**	**SNP in 5’ UTR**	**SNP in splicing site**	**SNP in STOP codon**
AG	Solyc02g071730.2.1	5	5074	426	3	0	3	0	0	0	0
ANT	Solyc04g077490.2.1	7	3097	1893	0	0	0	0	0	0	0
AGO1	Solyc03g098280.2.1	19	6274	2650	25	6	19	3	0	0	0
BAM1	Solyc02g091840.2.1	2	3625	3514	32	6	26	4	7	0	0
CLV1	Solyc04g081590.2.1	2	3926	2436	0	0	0	0	0	0	0
CLV2	Solyc04g056640.1.1	1	2240	2240	4	3	1	0	0	0	0
CNA	Solyc03g120910.2.1	14	6463	2109	15	4	11	2	0	0	0
FIL	Solyc01g091010.2.1	7	3194	1070	7	3	4	3	3	0	0
FW2.2	Solyc02g090730.2.1	3	669	610	5	1	4	2	0	0	0
KAPP	Solyc01g079720.2.1	8	14613	1118	9	4	5	2	0	0	0
KOR1	Solyc01g102580.2.1	6	3289	2509	0	0	0	0	0	0	0
LAS	Solyc07g066250.1.1	1	1286	1286	29	6	23	0	0	0	0
LC	Solyc02g083940.2.1	1	5663	164	1	0	1	0	0	0	0
OVATE	Solyc02g085500.2.1	2	1383	1186	2	2	0	3	0	0	1 (lost)
PHB	Solyc02g024070.2.1	15	5882	2362	9	4	5	0	2	0	0
PHV	Solyc08g066500.2.1	15	5274	2225	0	0	0	0	0	0	0
RBL	Solyc02g081680.2.1	14	4985	2650	0	0	0	0	0	0	0
REV	Solyc11g069470.1.1	18	5512	2508	43	5	38	0	0	1	0
SP/TFL1	Solyc06g074350.2.1	2	1929	510	5	2	3	2	0	0	0
SHD	Solyc04g081570.2.1	15	5102	2769	28	6	22	4	3	0	0
STM	Solyc02g081120.2.1	4	3426	1592	6	3	3	4	1	0	0
SYD	Solyc02g068560.2.1	4	14883	3334	44	22	22	1	0	0	0
SUN	Solyc10g079240.1.1	5	2229	1261	15	10	5	0	0	0	1 (gained)
TD380	Solyc02g085390.2.1	5	7707	577	0	0	0	0	0	0	0
TPL	Solyc03g117360.2.1	25	9952	3043	29	4	25	9	0	0	0
ULT1	Solyc07g054450.2.1	3	3273	699	4	1	3	3	2	0	0
UFO	Solyc02g081670.1.1	2	1367	1367	20	7	13	0	0	0	0
WUS	Solyc02g083950.2.1	3	1238	1003	0	0	0	0	0	0	0
ZLL	Solyc09g082830.2.1	18	6153	2652	37	8	29	0	0	1	0
**Supplementary genes within resequenced fragments**											
Adenylosuccinate synthetase	Solyc02g085520.2.1	1	8807	231	1	0	1	0	0	0	0
AHRD V1 - MDTK	Solyc02g090740.2.1	1	1608	1582	13	8	5	5	0	0	0
AHRD V1 - Q9LIL2_ARATH	Solyc01g102570.2.1	1	1314	62	0	0	0	0	0	0	0
ATP dependent RNA helicase	Solyc04g081580.2.1	2	6006	655	4	1	3	15	0	0	0
Glucosyltransferase	Solyc02g081690.1.1	1	1445	1445	11	4	7	0	0	0	0
Os01g0786800 protein	Solyc02g085400.2.1	3	2736	496	3	0	3	3	0	0	0
Ovate protein	Solyc02g085510.1.1	1	179	179	0	0	0	0	0	0	0

Effects are detected with SnpEff with tomato annotation ITAG 2.3 version.

### Population differentiation and structure

Pairwise F_ST_ on the whole genome dataset was low between SL and SLC (0.05%) while between SL and SP and SLC and SP a stronger differentiation was observed (1.6 and 2% respectively). SP and WT differentiation rose to 2% and average of SL vs WT and SLC vs WT to 5%. These results are supported by the STRUCTURE analysis on red fruit accessions output. Following Evanno’s deltaK correction, a two group’s population structure was identified as already obtained with a smaller set of SNP [37] (Additional file 2).

### Selection patterns across genetic groups

**Figure 3:**
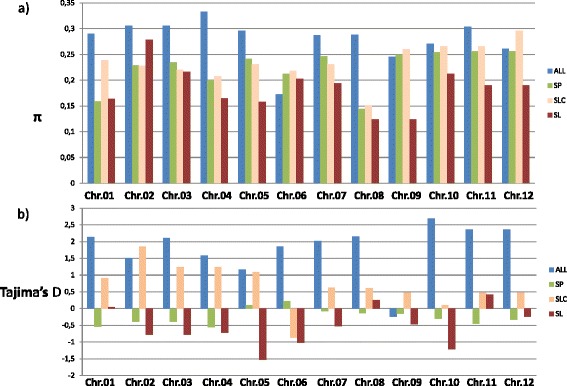
**Diversity revealed by genotyping 5,795 SNP of the SolCAP array. (a)** Chromosomal nucleotide diversity (π) and **(b)** Tajima’s D over the whole collection (ALL) and for 12 *S. pimpinellifolium* (SP), *63 S.l. cerasiforme* (SLC) and 17 *S. lycopersicum* (SL).

Nucleotide diversity (π estimates) and neutrality (Tajima’s D) were estimated first for each chromosome using the 7,667 SNPs of the SolCAP array (Figure 3) on 92 collection genotypes and related subgroups. Whole chromosome total nucleotide diversity ranged from 0.17 (chromosome 6) to 0.33 (chromosome 4) with an average genome-wide value of 0.27. Intraspecific values were estimated to 0.22, 0.23 and 0.18 for SP, SLC and SL, respectively. The ratio of total nucleotide diversity $\frac{\pi \kern0.5em s.\kern0.5em  lycopersicum}{\uppi \kern0.5em s.\kern0.5em  pimpinellifolium}$ was lower than 1 for all chromosomes but 1 and 2. Tajima’s D statistic was positive for all chromosomes but chromosome 9, with significant values for chromosomes 1, 2, 3, 5, 11 (P < 0.05) and a whole genome D value of 1.8. Intraspecific D was negative in SL (except on chromosomes 1, 8 and 11) -with lowest value on chromosome 5- and in SP (except on chromosomes 5 and 6). In SLC, D values were all positive except on chromosome 6.

**Figure 4:**
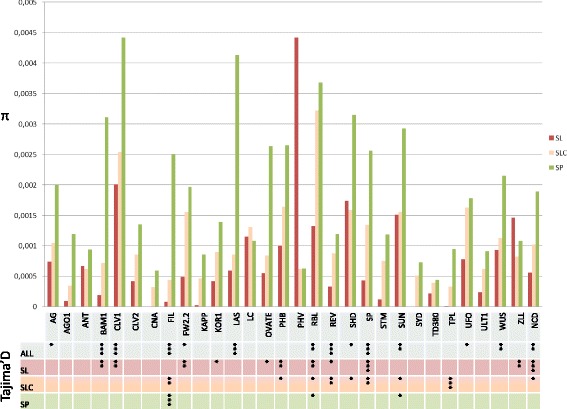
**Nucleotide diversity (π) in the 30 candidate genes for the three groups of 12** ***S. pimpinellifolium*** **(SP),** ***63 S.l. cerasiforme*** **(SLC) and 17** ***S. lycopersicum*** **(SL) accessions.** The genes for which Tajima’s D is significant in the 92 accessions collection are indicated (* = P-value < 0.05; ** = P-value < 0.01; *** = P-value < 0.001) as well as in SL, SLC and SP subgroups.

**Figure 5:**
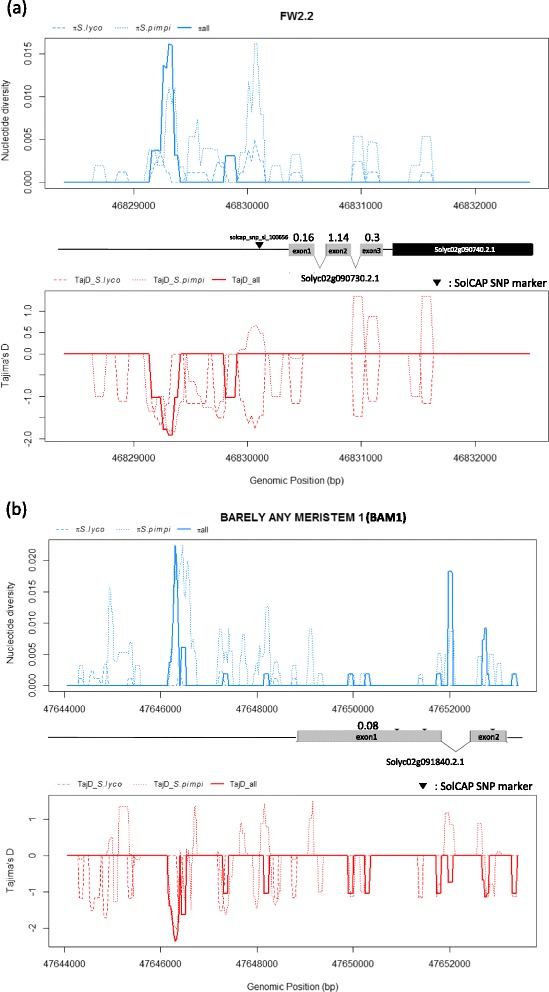
**Sliding-window analysis of nucleotide diversity (π) -and Tajima’s D according to genetic groups for FW.2.2 (a) and BAM1 (b) regions.** Gene annotation (ITAG 2.3) is displayed. Numbers above exons indicate d_N_/d_S_ values per exon.

Nucleotide diversity estimation, Tajima’s D and codon analysis (d_N_/d_S_) were then performed for the 30 re-sequenced fragments (Additional file 3). Nucleotide diversity between red-orange and green fruited species (*π*_*red_fruit_species*_ /π_*green_fruit_species*_*,* see Figure 2 for nomenclature) on re-sequenced data was low (0.10). Intraspecific nucleotide diversity estimates were the lowest for SL (π = 0.0007), followed by SLC (π = 0.001), SP (π = 0.002) and the wild types (π = 0.0120). Re-sequenced fragments showed low and heterogeneous nucleotide diversity, ranging from 1.65 × 10^−4^ (*FIL*) to 2 × 10^−6^ (*TPL*). Overall, D-statistic and π values followed a similar trend. Thirteen re-sequenced genes showed a significant D-statistic over the whole collection (Figure 4). The Tajima’s D analysis indicated significant evidence for selection in 15 fragments in at least one genetic group (11 fragments for SL, 9 for SLC and 4 for SP) as shown on Figure 5.

According to the codon based analysis, nine fragments showed a d_N_/d_S_ ratio significantly different from 1 (Table 3). The ratio was lower than one in all gene fragments, six genes showing dN/d_S_ ratio significantly different from 1 displayed also a significant D-statistic on the whole collection. To investigate further the candidates showing multiple signals, we performed a sliding-window approach for the aforementioned tests, allowing an exact positioning of the diversity/neutrality patterns along gene annotation. This allowed the identification of strong negative signals in upstream region of *FW2.2*, *BAM1, RBL, REV*, *CLV1*, as well as a positive signal in intragenic *OVATE* fragment. Overall, a contrasting pattern within intragenic sequence between SL and SP groups could be observed for *OVATE* and *FW2.2* (Figure 5 and Additional file 4).

**Table 3:** **Patterns of variation detected in the genes showing multiple selection signatures, based on 6106 SNP detected in 96 accessions (including wild accessions)**

	**CLV1**		**CNA**		**SYD**		**REV**		**FW2.2**		**RBL**		**BAM1**		**TD380**		**UFO**	
**Nucleotide diversity (π)**																		
SL	0.001559		0		0		0.000214		0.000283		0.000745		0.000175		0.000080		0.000518	
SLC	0.000022		0.000007		0.000050		0.000063		0.000015		0.000074		0.000036		0.000080		0.000217	
SP	0.001376		0.000079		0.000284		0.000548		0.000579		0.000638		0.001045		0.000171		0.000653	
WT	0.017814		0.004996		0.010979		0.010961		0.028111		0.011036		0.015765		0.013036		0.012120	
**Tajima’s D**																		
ALL	−2.566	***	−1.620	#	−1.554	n.s.	−2.515	***	−2.112	*	−2.214	**	−2.555	***	−1.528	n.s.	−1.897	*
SL	−2.177	**	n.a.	n.a.	n.a.	n.a.	−2.042	*	−2.205	**	−2.131	**	−2.302	**	−1.069	n.s.	−1.498	n.s.
SLC	−1.668	#	−1.077	n.s.	−1.207	n.s.	−2.455	**	−1.435	n.s.	−1.838	*	−1.784	#	−1.290	n.s.	−1.007	n.s.
SP	−0.856	n.s.	−1.429	n.s.	−1.288	n.s.	−0.844	n.s.	−1.553	n.s.	−1.903	*	−1.438	n.s.	−1.712	#	−1.490	n.s.
WT	−0.361	n.s.	−0.866	n.s.	0.041	n.s.	−0.465	n.s.	−0.201	n.s.	−0.459	n.s.	−0.708	n.s.	−0.119	n.s.	−0.538	n.s.
**Syn.-NonSyn. polymorphism**																		
dN	0.0076		0.0096		0.0235		0.0046		0.0135		0.0063		0.0013		0.0211		0.0152	
dS	0.0511		0.0286		0.0314		0.0228		0.0238		0.014		0.0178		0.0351		0.0317	
dN/dS	0.1550		0.3356		0.7529		0.2017		0.8074		0.4548		0.0774		0.6015		0.4794	

Nucleotide diversity (π), Tajima’s D and non synonymous (dN), synonymous (dS) and d_N_/d_S_ are shown. (n.s., not significant; #, P < 0.10; *, P < 0.05; **, P < 0.01; ***, P < 0.001).

### Genome-wide and candidate gene association

Phenotypic data (FW, LC) were previously described for the core collection [33]. Fruit shape index (FSI) was also assessed. Associations were detected by mixed linear model on the dataset including SNP from re-sequenced fragments and SolCAP array. Seven associations were identified after FDR corrections at a whole genome level (P < 0.05) involving SNP in 3 fragments and one SolCAP marker, all on chromosome 2 (Table 4). Locule number was associated with six closely linked markers and fruit shape index with only one. Two of these markers were previously identified as causal mutations in OVATE and *LC* on chromosome 2 [25,96].

**Table 4:** **List of associations detected on 90 accessions with locule number and fruit shape index**

**Phenotype**	**SNP_ID**	**Gene**	**Chromosome**	**SNP Position (bp)**	**p-value**
Locule number					
	solcap_snp_sl_23925		2	41345621	4.95 x10^−4^
	WUS_SNP_T_A_2117	WUS	2	41765967	1.28 x10^−3^
	LC_SNP_T_A_3774	LC	2	41765967	1.28 x10^−3^
	WUS_SNP_G_A_2168	WUS	2	41766018	1.07 x10^−2^
	WUS_SNP_T_C_3869	WUS	2	41767719	1.07 x10^−2^
	WUS_SNP_T_A_3979	WUS	2	41767829	4.95 x10^−4^
Fruit shape index					
	OVATE_SNP_A_G_269	OVATE	2	42944775	1.64 x10^−2^

P-values according to the false discovery rate procedure (FDR).

## Discussion

We successfully re-sequenced 30 large regions covering candidate loci involved in meristem development and maintenance or corresponding to fruit weight and shape QTL in 96 tomato accessions (92 red-fruited accessions and 4 distant species). We detected a total of 6,106 SNPs within these 30 candidate loci. We also genotyped 5,795 SNPs spread on the whole genome in 92 accessions. Within the wild (SP), admixed (SLC) and cultivated (SL) accessions, the analysis of the nucleotide diversity pattern resulted in two primary conclusions. First, admixed tomato maintained the largest amount of diversity within the collection. Second, the targeted genes showed in average a reduced diversity compared to whole genome values and several strong selection signatures were detected. Moreover, the investigation of selective footprints linked to domestication, in this set of 30 candidate genes related to meristem development, evidenced that a strong purifying selection is at play on this pathway. However the small sample size did not allow us to identify any new association for fruit traits within candidate genes nor in the whole genome data set.

### Polymorphism discovery

Among the studied taxa, polymorphism discovery showed considerable interspecific and intraspecific variations. Red fruited species and *S. cheesmaniae,* showed a drastic reduction of polymorphism compared to green fruited species, as already shown [110]. Overall, 3,747 SNPs were identified by mapping reads on the reference genome and 2,359 SNPs when using *de novo* assembly. Van Deynze and colleagues [111] estimated the nucleotide variation in conserved genes to 1 SNP per 1,627 bp in SLC, 1 per 5,675 bp in fresh market tomatoes and 1 per 851 bp for SP. Our results support these results for SLC (1/1,401 bp) but are sensibly different in SL (1/2,889 bp) and SP (1/406 bp). A possible explanation of this outcome is the difference in the plant material used, as SL and SP in Van Deynze and colleagues [111] are only represented by two and one accessions, respectively. Among the wild type accessions, *S. cheesmaniae* showed the lowest diversity (1 SNP every 1,297 bp). Together with pairwise F_ST_, these results support the previously established phylogeny of the Lycopersicon complex as well as the domestication scenario and its related bottlenecks [112-114]. Regarding coding mutations, important differences in the number of non-synonymous mutations were observed among candidates. The *OVATE* stop codon was identified as in [22] and could be related to fruit shape variation. SNP modifying splicing sites (*REV* and *ZLL*) may also alter the protein. Lack of polymorphism for some candidates suggested a strong selection pressure especially in meristem maintenance genes (*WUS*, *CLV1*).

### Nucleotide diversity and selection patterns across genetic groups

Nucleotide diversity ratio showed that rates of alterations varied between genes of the different meristem development pathway compartments with interesting features in the meristem maintenance genes. Intraspecific nucleotide diversity in the SL group is similar to values previously obtained by Labate and colleagues in European germplasm [115]. Over the panel, re-sequenced genes and flanking regions showed a similar profile, with a gradient loss from wild to cultivated species. Nevertheless a large range of variation remains between fragments (Figure 4).

Several significant deviations from the neutral expectation were detected by either analysis, the negative values of Tajima’s D and d_N_/d_S_ ratios smaller than 1 suggested purifying selection, especially on genes from the meristem maintenance compartment where six candidates showed significant D value (Table 3 and Additional file 3). Small sample size, low divergence among lineages and strength of positive selection affect the power of this kind of analysis. However, previous studies in plants suggested that strong purifying selection is a major player in plant genomes. Gossman and colleagues used a genome-wide approach to demonstrate that there is little evidence of adaptive evolution (through the accumulation of adaptive mutations) in many plant species [116]. One of the interpretations suggested by the authors is the small effective size of plant population (*Ne*), which implies that selection may have more impact on the fixation/loss of mutations. In tomato, Städler and colleagues [117] investigated the historical demography of wild tomatoes and demonstrated that the closest wild relative species exhibit concordant signatures of population-size reduction during the evolutionary history. In this context, our results are congruent with these previous observations.

In seven genes (*CLV1*, *FIL*, *LAS*, *TPL*, *REV*, *BAM1*, *SP*), the large and negative Tajima’s D test indicated an excess of rare nucleotide polymorphisms with low frequency compared with expectation under neutral theory. This could be explained by the effect of background selection [118], genetic hitchhiking [119] or by an extension of the effective population size (*Ne*) following a bottleneck. For example, *SP* and *LAS* have been previously characterized as key determinants for plant architecture, mutations in these genes inducing strong phenotypic modifications [54,60].

Nucleotide diversity analyses of genes associated with fruit morphology in plants have reported different evolutionary constrains related to gene function and gene fragments. In tomato, the fruit weight QTL *fw3.2* revealed reduced nucleotide diversity in SL and an overall reduced diversity compared with the entire chromosome. The corresponding gene, *SlKLUH*, showed significant local D values (positive and negative), supporting a selective pressure around the gene [120]. In the present study, candidate genes and their respective chromosomal Tajima’s D values were calculated and can be observed taking into account possible genotyping- sequencing platform ascertainment bias as previously observed in other species [121]. In a whole genome comparative transcriptome study of five tomato relatives, Koenig and colleagues identified only 51 genes showing d_N_/d_S_ >1 [114]. Regarding our gene set, the positive selection evidences underline the rareness of such events. Evolutionary variations of genes involved in traits such as seed/fruit morphology have been reported in other plant species. In *Arabidopsis*, the genetic robustness of cell cycle-related processes was found to be due to functional redundancy more than high selective constraint [29]. In potato, no significant deviation from neutrality was found for genes related to alkaloid pathway, d_N_/d_S_ ratios close to 1 and negative values of Tajima’s D test suggested purifying selection in the gene fragments [122].

### Differentiation and population structure

Pairwise F_ST_ analysis revealed variable trends of differentiation between sub-populations. If differentiation was low between SL and SLC, it was stronger for SL-SP and SLC-SP. These results are consistent with those described by Sim and colleagues [123] between cherry (SLC) and fresh market (SL) tomatoes. However, differentiation between SL and SP had a higher estimate in the aforementioned work. Lower differentiation may be explained by the low sample size of the SP group within our collection. The structure analysis detected two ancestral groups (SL and SP) and an admixture group composed mainly of SLC accessions. High correlation of the Q estimates (0.94, data not shown) with initial findings on the same panel using a smaller set of SNP markers is comforting results of Ranc and colleagues [33].

### A few mutations with an important role in fruit size variation

Genome wide association tests for three fruit traits revealed associations with SNP in two intervals surrounding previously described QTL for fruit shape and locule number on chromosome 2. Results from association highlighted two previously identified major loci accounting for fruit shape and size variation, namely *LC* and *OVATE.* We pinpointed the exact mutation of previously identified *LC* and *OVATE* genes (Table 4). We could not detect any other association, particularly with fruit weight, unless decreasing the statistic threshold. Together with the small sample size, a strong relationship between the population structure and fruit weight was shown, hampering the identification of consistent associations new for this trait. Nevertheless non neutral signals of evolution at loci underlying quantitative traits are expected to be different from those due to directional selection [124,125]. Ten genes showed multiple selection signals (Table 4). They include four genes involved in meristem maintenance (*BAM1*, *CLV1*, *REV* and *SYD*), three in floral meristem identity (*RBL*, *CAN* and *UFO*), and three genes previously detected in tomato for their role in phenotype (*TD380* as a main association with *FW*, *FW2.2* and *OVATE*).

For *FW2.2*, the major tomato fruit weight QTL, the analysis showed signals of selection, including important diversity loss between SL and SP taxa. Tajima’s D was strongly significant over the panel (−2.11) and remained significant in the SL group (−2.20). Similarly, d_N_/d_S_ ratio was close to 1 for the whole fragment and higher than 1 for exon 2, a cysteine-rich motif (24aa) part of a highly conserved core domain [91]. Tajima’s D sliding window analysis identified a strong negative peak within the promoter sequence of the *FW2.2* gene (Figure 5a). This finding is supported by the identification of an association signal by Knaap and colleagues in the same region [9]. Taken together, these clues will help to understand the mechanism underlying *FW2.2* regulation which is not yet unravelled. *BAM1* is a *CLAVATA1*-related Leucine rich repeat receptor-like kinases [88]. It is part of the CLAVATA regulation complex. It has been demonstrated that BAM genes play role in cell division by interacting with CLAVATA ligands in the meristem flanking regions [89,126]. *BAM1* has showed the most significant Tajima’s D (−2.55) among candidates and low d_N_/d_S_ (0.0774). Like in *FW2.2* region, a peak was observed in the gene upstream region (Figure 5b). This gene, located in a fruit weight QTL region should be further studied.

## Conclusions

Combining evolutionary metrics and quantitative genetic approach allowed us to decipher the genetic architecture of domestication traits and document their evolutionary history. We identified strong evidence of purifying selection within a few candidate genes with an emphasis on genes related to meristem maintenance. This loss of nucleotide diversity fits previously established domestication scenario [113,114]. Further experiments are required in two ways. The decreasing cost of sequencing will allow large scale GWAS experiments and selective sweep detection at the genome level in a very close future. This will help identifying new candidate loci. For the genes showing patterns of selection, expression profiling and fine scale studies such as methylation studies may uncover their regulation during fruit development as recently shown for the maturation process [127,128].

## Methods

### Selection of candidate genes

Candidate genes were selected following a three steps approach: literature review (1), sequence homology (2) and amplification success rate (3):

First, an extensive literature review identified 50 genes involved in meristem development in *Arabidopsis thaliana*. Related candidate gene protein sequences were extracted to identify their orthologs in tomato. Orthologs were obtained from NCBI database (www.ncbi.nlm.nih.gov*)* using TBLASTN procedure (Additional file 5). The output data was sorted according to e-values and bit score. Candidate genes without a match were screened using TBLASTN on the tomato scaffolds genome assembly (v2.40) (see step (2) in the Additional file 1). Reciprocal BLASTN between query and subjects was performed to support the similarity (Additional file 6). For all orthologous sequences, a BLASTN was used to identify their corresponding candidate genes in the tomato genome including the flanking promoter and 3’ UTR sequences. Final selection was based on amplification success rate (>90 individuals amplified) and specificity (single PCR product).

### Plant materials

A total of 96 accessions (Additional file 7) were selected to represent the maximum diversity of a larger collection drawn from 360 accessions previously described in [37]. The set was composed of 63 *S.l. cerasiforme* (SLC); 12 *S. pimpinellifolium* (SP); 17 *S. lycopersicum* (SL) (including Heinz1706, the reference sequenced genome) and four wild relatives (WT) *S. pennellii* (LA716), *S. habrochaites* (PI247087), *S. chmielewskii* (LA1840) *and S. cheesmaniae* (LA1401). Accessions were derived from French researchers’ prospecting, breeders’ collections, the Tomato Genetics Resource Center (Davis, USA), the Centre for Genetic Resources (Wageningen, The Netherlands), the North Central Regional Plant Introduction Station (Ames, IA) and the N.I. Vavilov Research Institute of Plant Industry (St. Petersburg, Russia). Accessions are characterized and maintained at INRA, Avignon, France. Phenotyped traits (FW and locule number) data were collected from [33]. Tomato Analyzer V2.1.0.0 software [129] was implemented to scan fruit morphology within the 96 accessions. Then, fruit shape index (FSI, ratio of maximum diameter/height) was analyzed. For each the three phenotypic traits, year and accession effect were statistically corrected using Anova using the [R] software (www.R-project.org). Adjusted mean was calculated by “all.effects” procedure package implemented in [R].

### DNA isolation and sequencing

Genomic DNA was isolated from 100 mg of frozen leaves using the DNeasy Plant Mini Kit (QIAGEN, Valencia, CA) according the manufacturer’s recommendations. DNA titration was performed using fluorescence. We used long range PCR (LR-PCR) to amplify DNA sequences (5-10 kb) and cover candidate genes and their potential regulatory regions. Amplification primers were designed in Primer3 (http://www.bioinformatics.nl/cgi-bin/primer3plus/primer3plus.cgi/), see Additional file 8 for a list of oligonucleotides. Amplification reactions were performed in a final volume of 50 μL in a reaction mix composed of 10 ng of template DNA, 10 pmol of each primer, 100 mM concentration of each deoxynucleotide, 5X Taq polymerase buffer P, and 1 unit of Taq polymerase Herculase II (Agilent, CA, USA). After 5 min of denaturation at 95°C, 35 cycles were performed with initial denaturation (20 s at 95°C), annealing during 20 s at 58°C, extension during 6 min at 68°C, followed by a final extension step of 8 min at 68°C. All PCR amplifications were checked on agarose gel (1%, 120 mV, 40 min). All successful and specific PCR products were selected and quantified using Quant’it picogreen dsDNA Assay kit (Invitrogen, Eugene, Oregon, USA) on a fluorescent plate reader (Perkin Elmer 2103 Multilabel reader). Pairs of primers revealing single-band polymerase chain reaction (PCR) product were chosen for sequencing. The thirty PCR fragments were pooled by accession in equimolar quantity. The DNA concentration of each pool was then adjusted to a final concentration of 0.5 μM (in a 100 μl final volume). These 96 pools were used to obtain the corresponding 454 libraries.

Each DNA library was fragmented by high pressure nitrogen flow to a 300-500 bp size range [130]. Fragmentation quality assessment was performed on an Agilent Bioanalyzer (Agilent technologies, USA). Each library was tagged using a specific sequence tag (GS Rapid Library Prep Kit, Roche diagnostics, Basel, Switzerland). Sequencing experiment was defined as followed: on the 454 sequencing picotiter plate, 8 regions (gaskets), each one containing 12 pools, each pool identified with a specific sequence tag [131]. Serial dilution and fine quantification was performed with Biomark Slingshot method (Fluidigm, San Francisco, California, USA). Emulsion-based amplification, GS-FLX library sequencing performed as described by Margulies [132]. Library preparation and 454 GS-FLX pyrosequencing (Roche diagnostics, 454 life science corp., Brandford, Connecticut, USA) were performed at Genotoul Genomic (http://www.genotoul.fr, INRA Toulouse, France).

### Read mapping, *de novo* assembly and polymorphism discovery

Checking for contaminants and quality trimming was performed using PyroCleaner software suite [133]. Assembly and polymorphism discovery were performed using NGen® version 3 (DNASTAR, Madison, WI, USA) [134]. Reads were mapped on the reference genome V2.4 from the Solanaceae Genomics Network (http://solgenomics.net/organism/Solanum_lycopersicum/genome). To improve mapping accuracy of wild accessions, a *de novo* assembly was performed using a BLAST-like Alignment Tool (BLAT) procedure [135]. Genome annotation 2.3 version produced by the International Tomato Annotation Group (http://solgenomics.net/organism/Solanum_lycopersicum/genome) was used to predict gene sequence architecture. We used SnpEff [136] to classify polymorphisms into non coding or coding polymorphisms (either synonymous or non-synonymous). Genes were also checked for open reading frame using ORF finder (http://www.ncbi.nlm.nih.gov/projects/gorf/). The longest ORF were kept for subsequent analysis. Polymorphisms were selected with a minimal coverage of 10x and polymorphism occurrence higher than 90%.

### Whole genome genotyping using the SolCAP array

Whole genome SNP genotyping was performed using Infinium assay (Illumina) developed by the Solanaceae Coordinated Agricultural Project -SolCAP- [109,137] as described in [138]. Probe sequences and related information are available from SolCAP (http://solcap.msu.edu). The SNP calling rate threshold per locus was set to 90%. Among the 8,784 SNPs from the SolCAP array, 7,667 SNPs passed the quality control. This SNP dataset –without Minor Allele Frequency filtering (MAF) was considered as a neutral dataset, a comparative basis for the candidate genes. To perform GWAS, filtering for low MAF (10% threshold) and missing data (10%), 5,795 SNPs were performed.

### Estimation of population differentiation, structure and relatedness

Sequencing and genotyping data on the collection of 96 accessions were subjected to genetic diversity indices calculation. Total nucleotide diversity (π) and Tajima’s D test [139] were computed on the collection and genetic subgroups (SL, SLC and SP) using DNAsp [140] and Variscan [141] software, using global calculation per chromosome for SolCAP array and sliding window on re-sequenced genes. On re-sequencing data, the d_N_/d_S_ neutrality test was performed on the synonymous (d_N_) to non-synonymous (d_S_) substitution rates [142,143] using PAML [144] with the YN00 module and a neighbor joining phylogenetic tree calculated on genotyping dataset to calibrate the d_S_ (Additional file 9). This ratio provides insights of selective pressures acting on protein-coding regions and allows identifying positive selection (d_N_/d_S_ >1) or purifying selection (d_N_/d_S_ <1). The pairwise-population fixation differentiation index, F_st_ [145], was assessed on the core collection between the three groups of accessions SL, SLC and SP and an outgroup constituted of the four wild types.

Population stratification (Q matrix) was defined with STRUCTURE [146] and Evanno’s correction [147] using the whole genome genotyping data. Simulations were ran with group number ranging from K = 1 to K = 10. Ten replicates (burnin period: 100,000 and MCMC step: 500,000) using the Bioportal computing cluster (http://app3.titan.uio.no/) with parameters as described in [148] Pairwise kinship coefficient calculation matrix (K matrix) was performed using Spagedi [149] following the Ritland calculation method [150].

### GWA mapping

Associations between polymorphisms and adjusted means of fruit traits were screened with TASSEL v3.0 [151]. For each trait, a mixed linear model (MLM) accounting for kinship (K matrix) and population structure (Q matrix) was used. Significance levels for multiple tests was corrected using FDR [152] correction method with 5% threshold.

### Data availability

The SNP genotype and phenotype datasets as well as *ad hoc* Q and K matrixes are deposited on the GNPis repository hosted https://urgi.versailles.inra.fr/association/association/viewer.do#panelCard/id=3 [153].

## Additional files

Additional file 1:
**Physical location of the candidate genes onto the tomato genome.** All sequences have been aligned on the tomato genome (v2.40) using BLAST. The distances are indicated in Mb. Additional file 2:
**Structure graphical outputs on 90 accessions based on different genotypic datasets.** (a) SolCAP genotyping data; b) re-sequenced genotypic data; (c) SNP data from Xu *et al.* 2012). Fruit weight variation is displayed in black line. Additional file 3:
**Nucleotide diversity (π), Tajima’ D and d**_**N**_ **/d**_**S**_ **for each candidate, according to the genetic groups: whole collection, SP, SLC and SL groups.**
Additional file 4:
**Sliding window analysis of nucleotide diversity (π) -and Tajima’s D according to genetic groups for CLV1 (a), RBL (b), REV (c) and OVATE (d) regions.** Gene annotation (ITAG 2.3) is displayed. Numbers above exons indicate d_N_/d_S_ values per exon. Red ‘*’ indicates the presence of a STOP codon. Additional file 5:
**Pipeline used for orthologous gene identification from** ***A. thaliana*** **to** ***S. lycopersicum.***
Additional file 6:
**List of Arabidopsis candidate genes proteins (TAIR 10).** Output of the TBLASTn on tomato genome sequence (v2.40). Additional file 7:
**Information on the 96 accessions used in the association study and diversity analysis: List of accessions, subgroups and phenotypic data.** Species are indicated as well as values for fruits weigh (FW), locule number (LCN) and fruit shape index (FSI). Additional file 8:
**Primer list for re-sequenced candidates (v.2.40).**
Additional file 9:
**NJ tree (1000 bootstrap) on 96 accessions.** In red, *S. lycopersicum* (SL) accessions; in orange, *S. lycopersicum* var. *cerasiforme* (SLC) accessions; in green: *S. pimpinellifolium* (SP) accessions. In black, outgroup formed of four wild species accessions (WT): *S. chmielewskii, S. habrochaites, S. peruvianum and S. pennellii*.
