# Supplementary figures and images for: Genes involved in floral meristem in tomato exhibit drastically reduced genetic diversity and signature of selection
Source: BMC Plant Biol. 2014 Oct 19;14:279. doi: 10.1186/s12870-014-0279-2 (PMC4210547; doi:10.1186/s12870-014-0279-2)

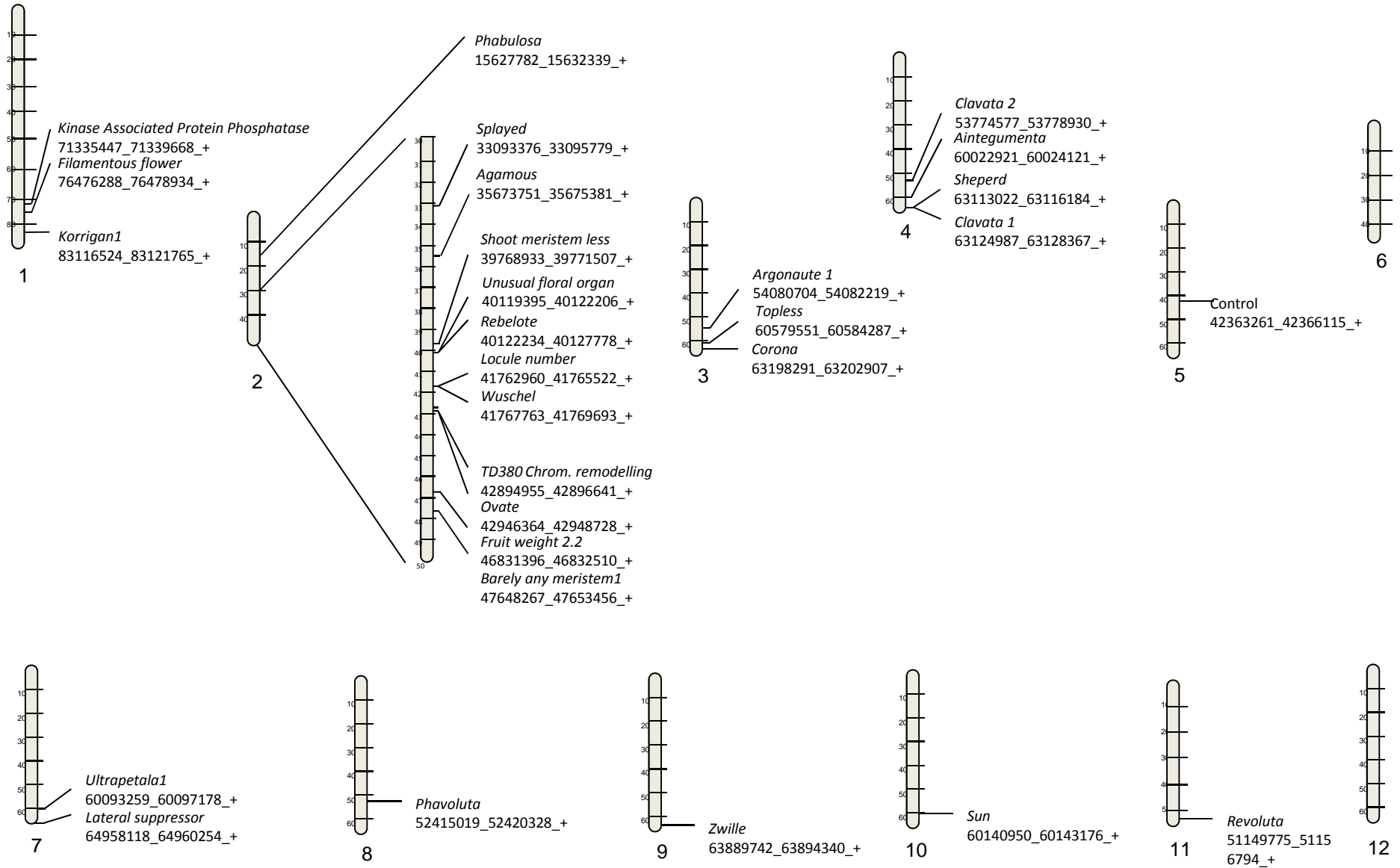

Supplement: Additional file 1: — Physical location of the candidate genes onto the tomato genome. All sequences have been aligned on the tomato genome (v2.40) using BLAST. The distances are indicated in Mb. [file 12870_2014_279_MOESM1_ESM.pdf]

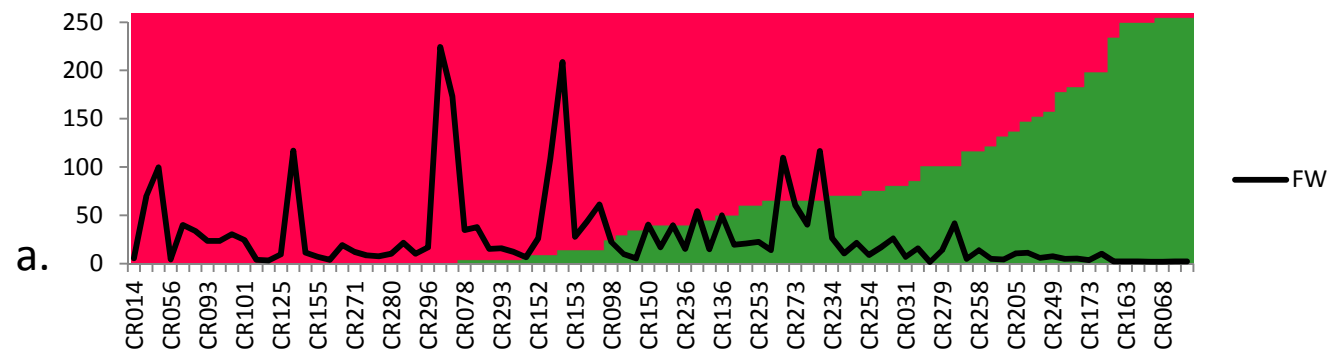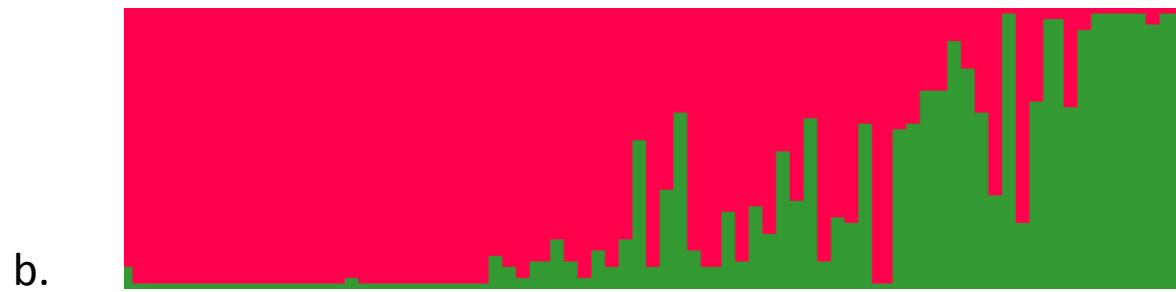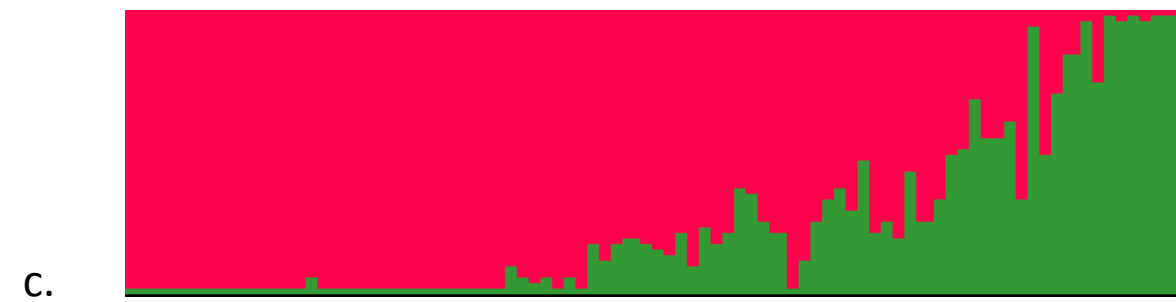

Supplement: Additional file 2: — Structure graphical outputs on 90 accessions based on different genotypic datasets. (a) SolCAP genotyping data; b) re-sequenced genotypic data; (c) SNP data from Xu et al. 2012). Fruit weight variation is displayed in black line. [file 12870_2014_279_MOESM2_ESM.pdf]

# CLAVATA 1

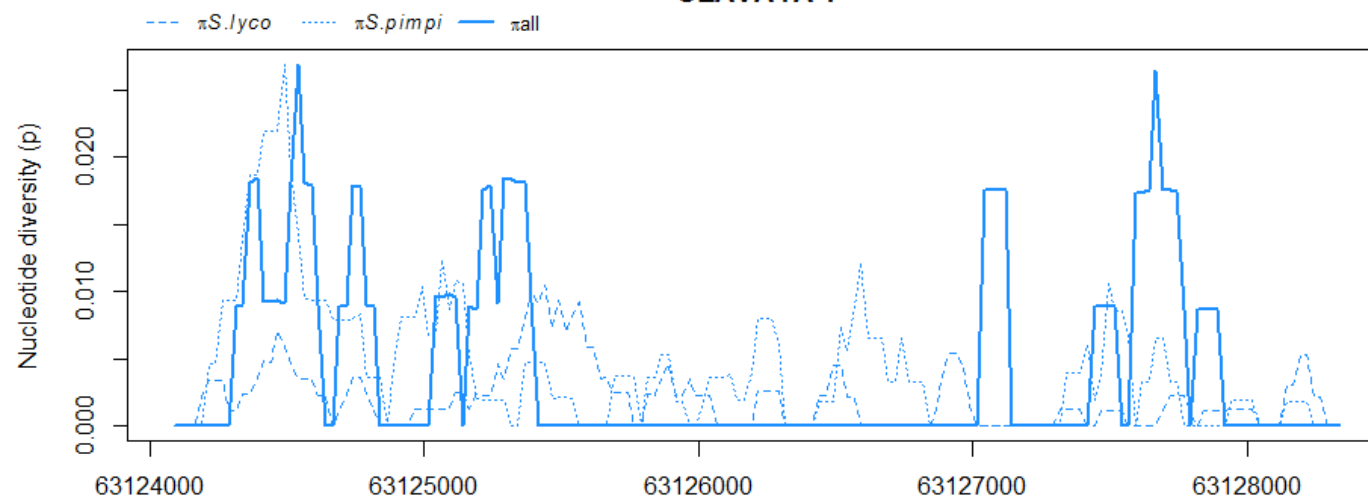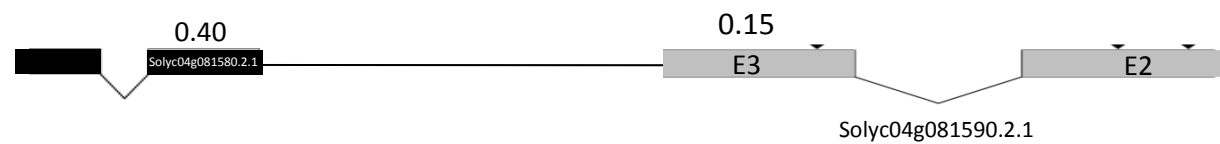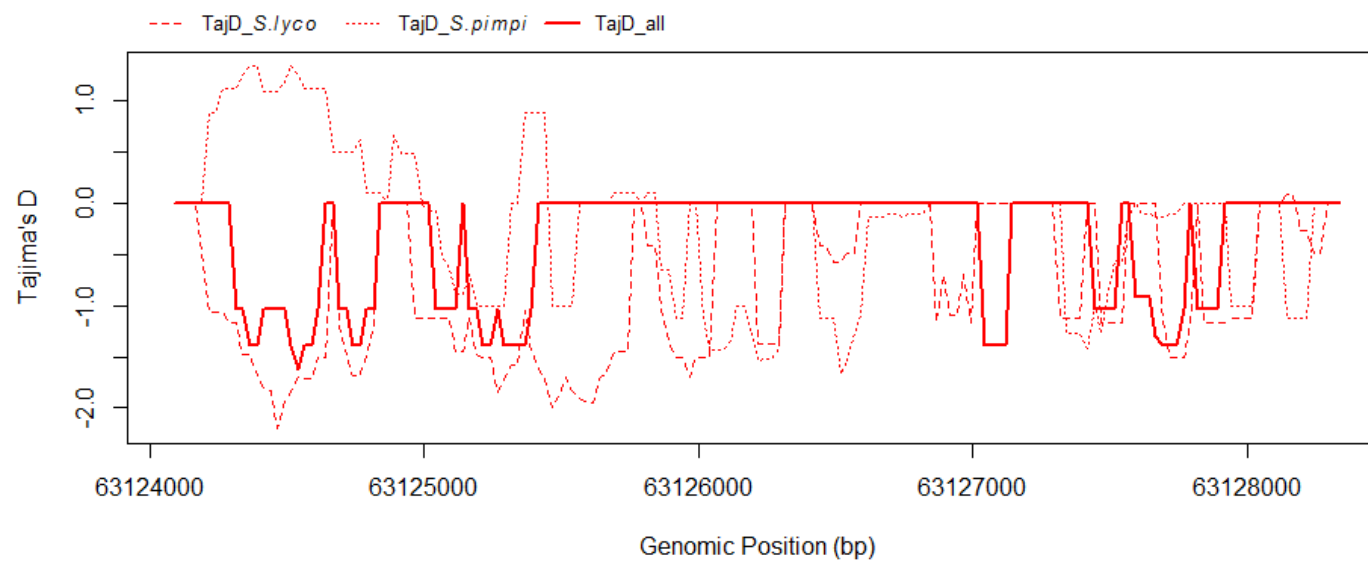

# REVOLUTA

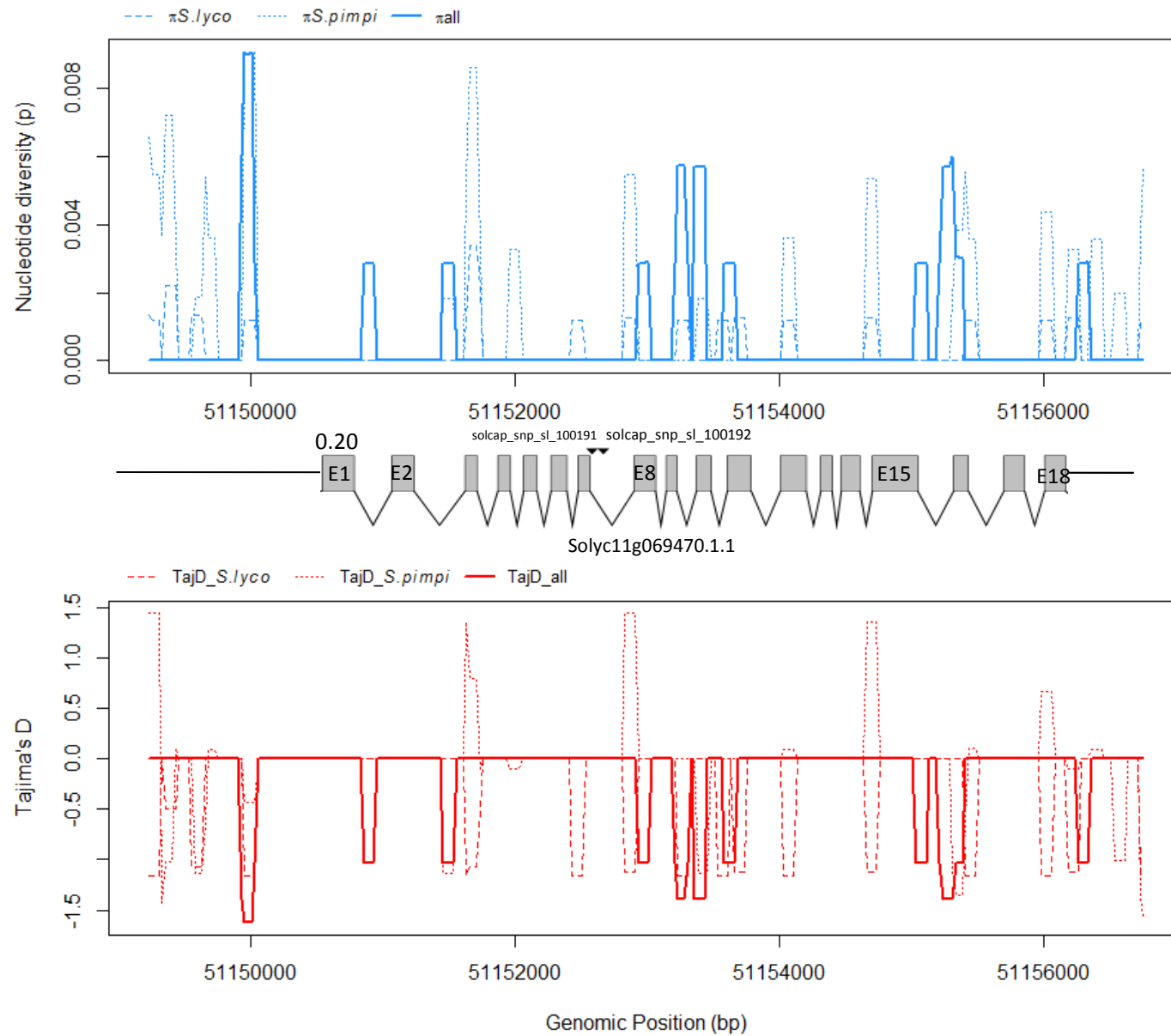

# REBELOTE

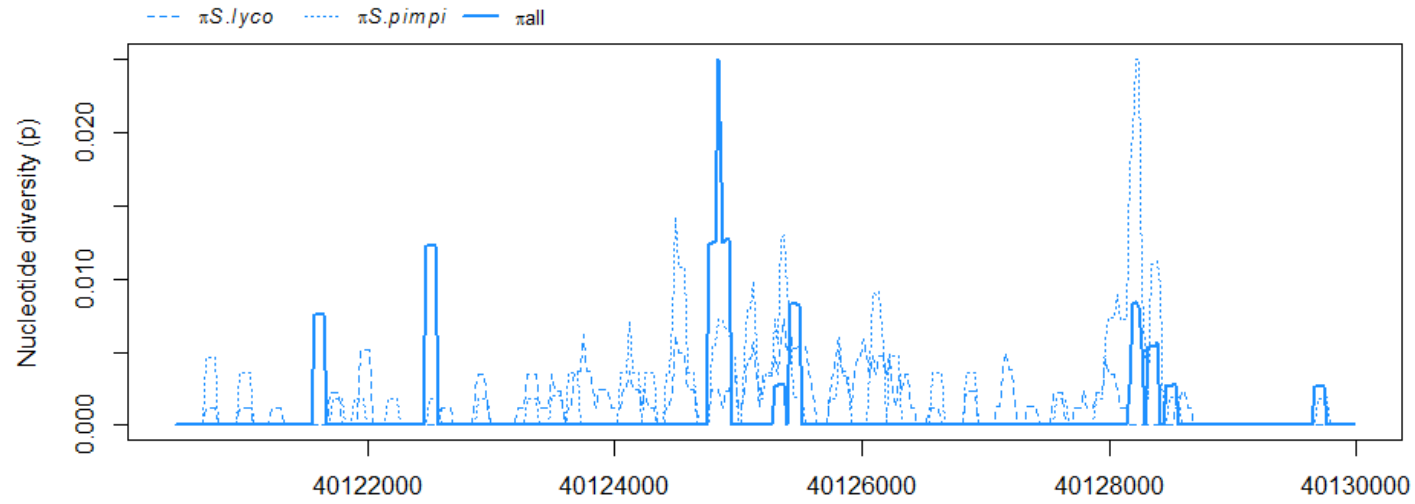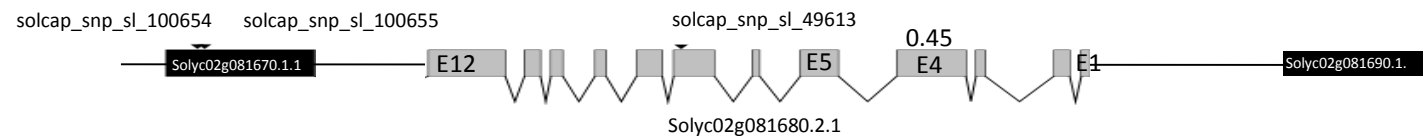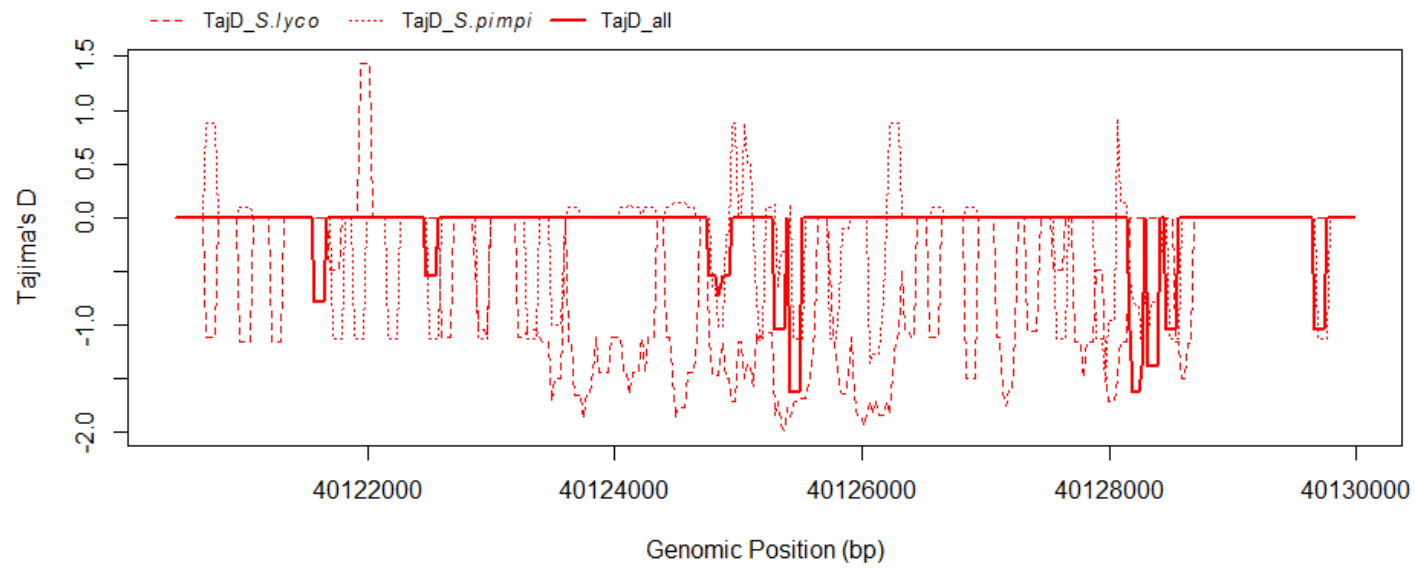

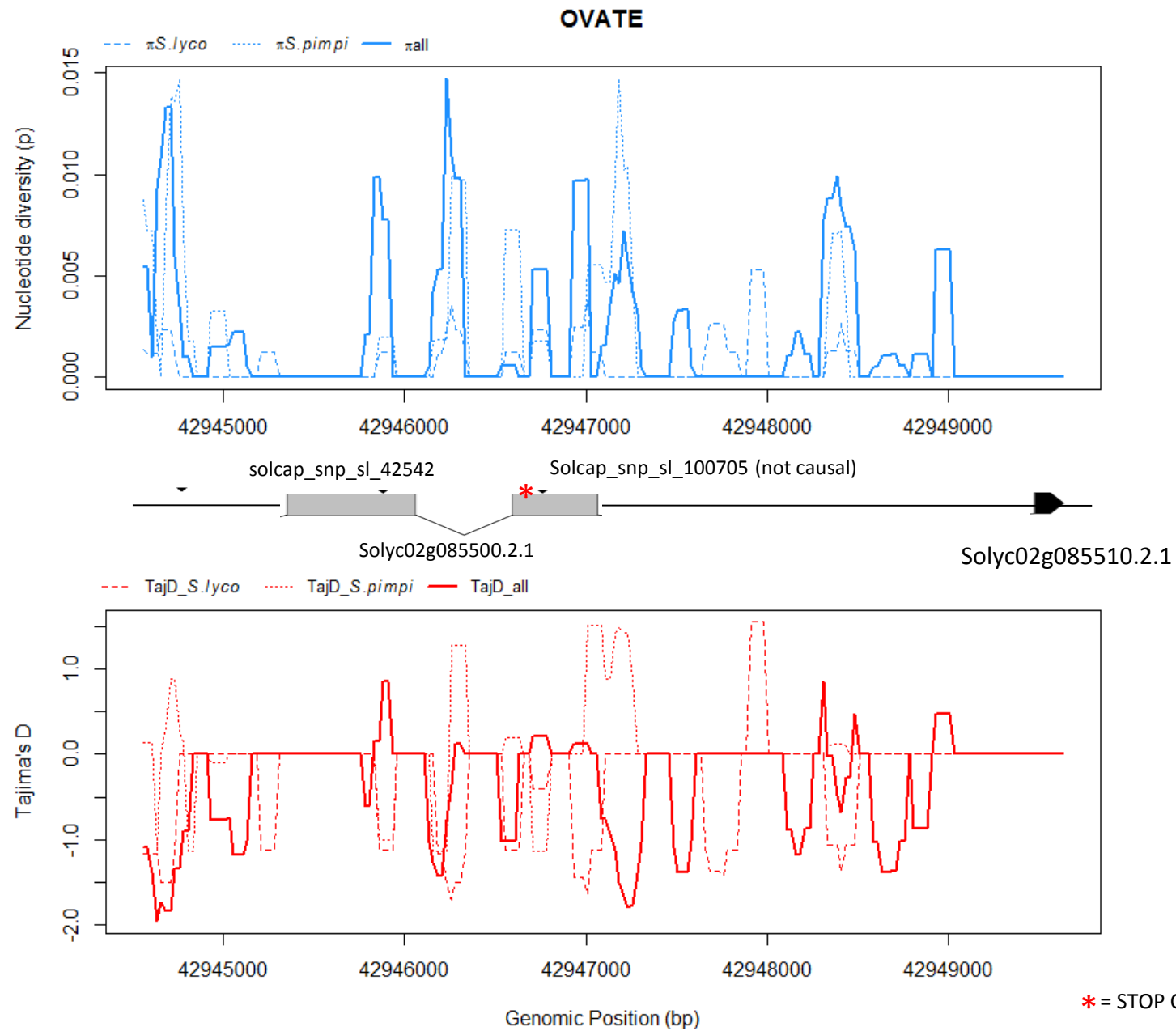

Supplement: Additional file 4: — Sliding window analysis of nucleotide diversity (π) -and Tajima’s D according to genetic groups for CLV1 (a), RBL (b), REV (c) and OVATE (d) regions. Gene annotation (ITAG 2.3) is displayed. Numbers above exons indicate dN/dS values per exon. Red ‘*’ indicates the presence of a STOP codon. [file 12870_2014_279_MOESM4_ESM.pdf]

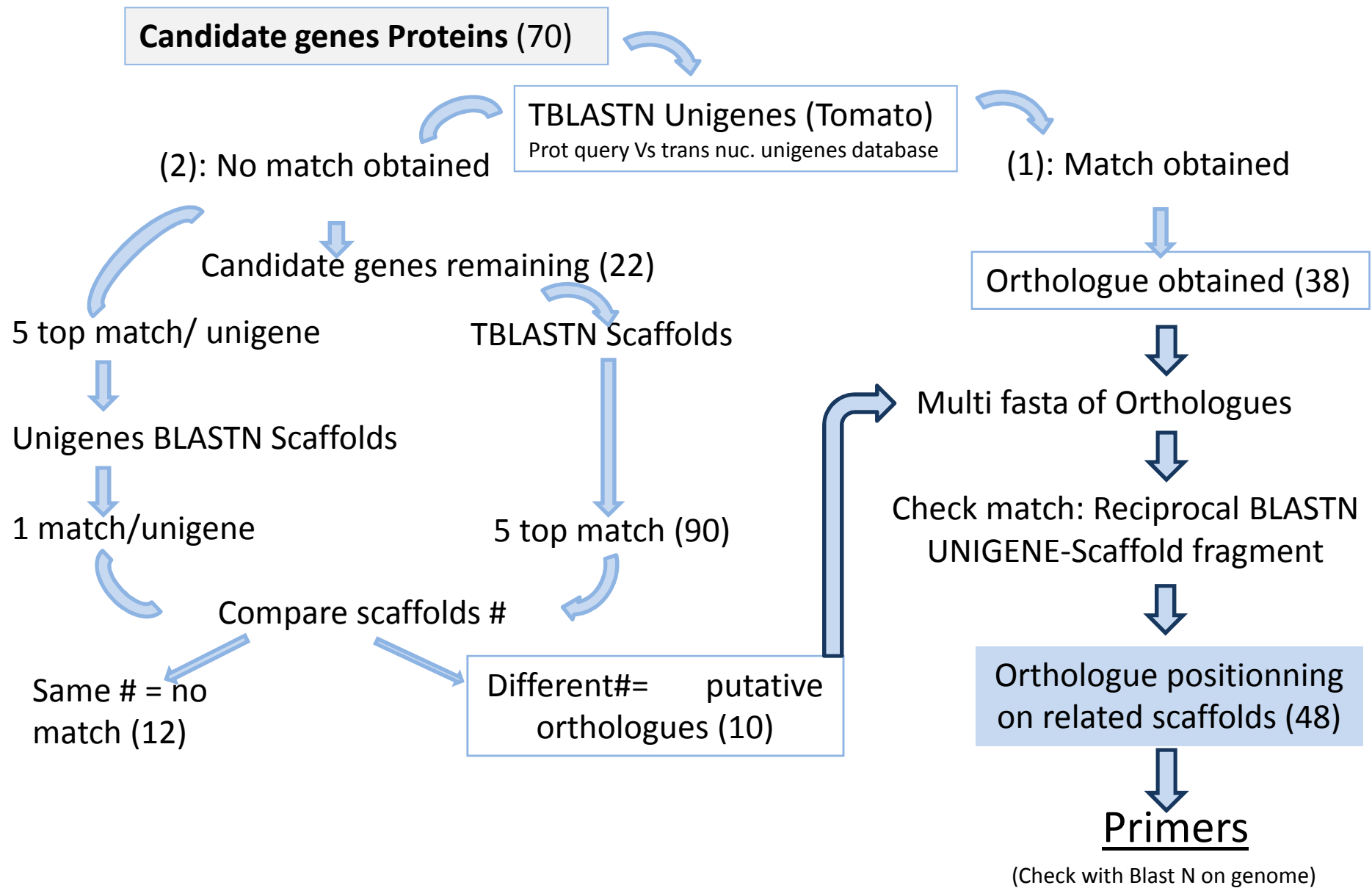

Supplement: Additional file 5: — Pipeline used for orthologous gene identification from A. thaliana to S. lycopersicum. [file 12870_2014_279_MOESM5_ESM.pdf]

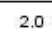

Supplement: Additional file 9: — NJ tree (1000 bootstrap) on 96 accessions. In red, S. lycopersicum (SL) accessions; in orange, S. lycopersicum var. cerasiforme (SLC) accessions; in green: S. pimpinellifolium (SP) accessions. In black, outgroup formed of four wild species accessions (WT): S. chmielewskii, S. habrochaites, S. peruvianum and S. pennellii. [file 12870_2014_279_MOESM9_ESM.pdf]
