# Supplementary material for: Genes involved in floral meristem in tomato exhibit drastically reduced genetic diversity and signature of selection
Source: BMC Plant Biol. 2014 Oct 19;14:279. doi: 10.1186/s12870-014-0279-2 (PMC4210547; doi:10.1186/s12870-014-0279-2)
Supplement: Additional file 3: — Nucleotide diversity (π), Tajima’ D and d N /d S for each candidate, according to the genetic groups: whole collection, SP, SLC and SL groups. [file 12870_2014_279_MOESM3_ESM.docx]

| **Additional file 3: List of nucleotide diversity (π), Tajima’ D, dN/dS and for each candidate, according to genetic groups: whole collection, SP, SLC and SL groups.** | | | | | | | | | | | | | |
| --- | --- | --- | --- | --- | --- | --- | --- | --- | --- | --- | --- | --- | --- |
| **Fragment_ID** | **Taxa_group** | **Ingroup** | **Sites** | **NetSites** | **Pi** | **Pi pw** | **Pi column** | **TajimaD** | **SigD** | **d_N_** | **d_S_** | **d_N_/d_S_** | |
| CLV1 | ALL | 92 | 4328 | 4179 | 0.000104 |  |  | -2.5665 | *** | 0.00768775510204082 | 0.0511938775510204 | 0.155072923210435 | |
|  | SLC | 64 | 4328 | 4189 | 0.000022 | 0.00254 | 0.00283 | -1.6681 | # | n.a. | n.a. | n.a. | |
|  | SL | 17 | 4328 | 4254 | 0.001559 | 0.00201 | 0.00209 | -2.1773 | ** | n.a. | n.a. | n.a. | |
|  | SP | 11 | 4328 | 4228 | 0.001376 | 0.00442 | 0.00556 | -0.8568 | n.s. | n.a. | n.a. | n.a. | |
|  | WT | 4 | 4328 | 4257 | 0.017814 | 0.02049 | 0.02371 | -0.3618 | n.s. | n.a. | n.a. | n.a. | |
| CNA | ALL | 92 | 4617 | 4562 | 0.000014 |  |  | -1.6203 | # | 0.0096 | 0.0286 | 0.335664335664336 | |
|  | SLC | 64 | 4617 | 4565 | 0.000007 | 0.00032 | 0.00043 | -1.077 | n.s. | n.a. | n.a. | n.a. | |
|  | SL | 17 | 4617 | 4579 | 0 | 0 | 0 | n.a. | n.a. | n.a. | n.a. | n.a. | |
|  | SP | 11 | 4617 | 4592 | 0.000079 | 0.00059 | 0.00161 | -1.4296 | n.s. | n.a. | n.a. | n.a. | |
|  | WT | 4 | 4617 | 4604 | 0.004996 | 0.00567 | 0.0065 | -0.8666 | n.s. | n.a. | n.a. | n.a. | |
| FIL | ALL | 92 | 5562 | 5414 | 0.000165 |  |  | -2.796 | *** | 0 | 0 | 0 | |
|  | SLC | 64 | 5562 | 5417 | 0.000052 | 0.00044 | 0.00053 | -2.1766 | ** | n.a. | n.a. | n.a. | |
|  | SL | 17 | 5562 | 5544 | 0.00008 | 0.00008 | 0.00008 | 0.9498 | n.s. | n.a. | n.a. | n.a. | |
|  | SP | 11 | 5562 | 5481 | 0.001327 | 0.0025 | 0.00451 | -2.1919 | *** | n.a. | n.a. | n.a. | |
|  | WT | 4 | 5562 | 5513 | 0.018381 | 0.01917 | 0.0199 | -0.6475 | n.s. | n.a. | n.a. | n.a. | |
| KAPP | ALL | 92 | 6747 | 6625 | 0.000007 |  |  | -1.3893 | n.s. | 0 | 0 | 0 | |
|  | SLC | 64 | 6747 | 6628 | 0.000022 | 0.00047 | 0.00061 | -0.3894 | n.s. | n.a. | n.a. | n.a. | |
|  | SL | 17 | 6747 | 6704 | 0 | 0.00002 | 0.00002 | n.a. | n.a. | n.a. | n.a. | n.a. | |
|  | SP | 11 | 6747 | 6632 | 0.000082 | 0.00086 | 0.00167 | -1.6 | # | n.a. | n.a. | n.a. | |
|  | WT | 4 | 6747 | 6714 | 0.008937 | 0.01001 | 0.01107 | -0.3673 | n.s. | n.a. | n.a. | n.a. | |
| LAS | ALL | 92 | 5047 | 4927 | 0.00015 |  |  | -2.7654 | *** | 0 | 0 | 0 | |
|  | SLC | 64 | 5047 | 4927 | 0 | 0.00086 | 0.00128 | n.a. | n.a. | n.a. | n.a. | n.a. | |
|  | SL | 17 | 5047 | 5000 | 0.000468 | 0.00059 | 0.00065 | -1.2807 | n.s. | n.a. | n.a. | n.a. | |
|  | SP | 11 | 5047 | 5015 | 0.003227 | 0.00413 | 0.00456 | -1.7551 | # | n.a. | n.a. | n.a. | |
|  | WT | 4 | 5047 | 4991 | 0.014693 | 0.01566 | 0.01766 | -0.6953 | n.s. | n.a. | n.a. | n.a. | |
| PHB | ALL | 92 | 5333 | 5293 | 0.00012 |  |  | -1.7529 | # | 0 | 0 | 0 | |
|  | SLC | 64 | 5333 | 5299 | 0.000135 | 0.00164 | 0.00183 | -1.8155 | * | n.a. | n.a. | n.a. | |
|  | SL | 17 | 5333 | 5307 | 0.0002 | 0.001 | 0.00119 | -2.1709 | ** | n.a. | n.a. | n.a. | |
|  | SP | 11 | 5333 | 5319 | 0.002065 | 0.00265 | 0.00288 | 0.6871 | n.s. | n.a. | n.a. | n.a. | |
|  | WT | 4 | 5333 | 5304 | 0.009867 | 0.0114 | 0.01294 | -0.8105 | n.s. | n.a. | n.a. | n.a. | |
| PHV | ALL | 92 | 5994 | 5961 | 0.000004 |  |  | -1.0371 | n.s. | 0 | 0 | 0 | |
|  | SLC | 64 | 5994 | 5961 | 0 | 0.00062 | 0.00078 | n.a. | n.a. | n.a. | n.a. | n.a. | |
|  | SL | 17 | 5994 | 5968 | 0.000059 | 0.00442 | 0.00556 | -1.7057 | # | n.a. | n.a. | n.a. | |
|  | SP | 11 | 5994 | 5963 | 0.000061 | 0.00063 | 0.00107 | -1.4296 | n.s. | n.a. | n.a. | n.a. | |
|  | WT | 4 | 5994 | 5974 | 0.000753 | 0.00075 | 0.00075 | -0.8294 | n.s. | n.a. | n.a. | n.a. | |
| SHD | ALL | 92 | 5953 | 5844 | 0.000033 |  |  | -2.1518 | * | 0 | 0 | 0 | |
|  | SLC | 64 | 5953 | 5847 | 0.000067 | 0.00159 | 0.00201 | -2.0053 | * | n.a. | n.a. | n.a. | |
|  | SL | 17 | 5953 | 5917 | 0.001067 | 0.00174 | 0.00194 | -1.5258 | n.s. | n.a. | n.a. | n.a. | |
|  | SP | 11 | 5953 | 5892 | 0.000667 | 0.00315 | 0.00437 | -1.6234 | # | n.a. | n.a. | n.a. | |
|  | WT | 4 | 5953 | 5921 | 0.004419 | 0.00553 | 0.00664 | 0.4458 | n.s. | n.a. | n.a. | n.a. | |
| STM | ALL | 92 | 5452 | 5407 | 0.000032 |  |  | -1.7832 | # | 0 | 0 | 0 | |
|  | SLC | 64 | 5452 | 5409 | 0.000082 | 0.00075 | 0.00099 | -1.0265 | n.s. | n.a. | n.a. | n.a. | |
|  | SL | 17 | 5452 | 5435 | 0 | 0.00012 | 0.00017 | n.a. | n.a. | n.a. | n.a. | n.a. | |
|  | SP | 11 | 5452 | 5407 | 0.000101 | 0.00118 | 0.00225 | -1.6 | # | n.a. | n.a. | n.a. | |
|  | WT | 4 | 5452 | 5419 | 0.001107 | 0.0019 | 0.00294 | -0.8405 | n.s. | n.a. | n.a. | n.a. | |
| SYD | ALL | 92 | 4892 | 4824 | 0.000018 |  |  | -1.5545 | n.s. | 0.0235743772241993 | 0.0314330960854092 | 0.752987678900461 | |
|  | SLC | 64 | 4892 | 4827 | 0.00005 | 0.00051 | 0.00054 | -1.2075 | n.s. | n.a. | n.a. | n.a. | |
|  | SL | 17 | 4892 | 4881 | 0 | 0 | 0 | n.a. | n.a. | n.a. | n.a. | n.a. | |
|  | SP | 11 | 4892 | 4873 | 0.000284 | 0.00073 | 0.00088 | -1.2884 | n.s. | n.a. | n.a. | n.a. | |
|  | WT | 4 | 4892 | 4888 | 0.010979 | 0.01125 | 0.01152 | 0.0415 | n.s. | n.a. | n.a. | n.a. | |
| TPL | ALL | 92 | 9086 | 8798 | 0.000002 |  |  | -1.0371 | n.s. | 0 | 0 | 0 | |
|  | SLC | 64 | 9086 | 9069 | 0.000089 | 0.00033 | 0.00038 | -2.6407 | *** | n.a. | n.a. | n.a. | |
|  | SL | 17 | 9086 | 8798 | 0 | 0.00001 | 0.00001 | n.a. | n.a. | n.a. | n.a. | n.a. | |
|  | SP | 11 | 9086 | 9077 | 0.000549 | 0.00095 | 0.00108 | -2.0191 | ** | n.a. | n.a. | n.a. | |
|  | WT | 4 | 9086 | 9065 | 0.016455 | 0.01704 | 0.0177 | -0.3891 | n.s. | n.a. | n.a. | n.a. | |
| ULT1 | ALL | 92 | 5525 | 5460 | 0.000016 |  |  | -1.222 | n.s. | 0 | 0 | 0 | |
|  | SLC | 64 | 5525 | 5461 | 0.000017 | 0.00062 | 0.00089 | -0.7214 | n.s. | n.a. | n.a. | n.a. | |
|  | SL | 17 | 5525 | 5469 | 0 | 0.00024 | 0.00031 | n.a. | n.a. | n.a. | n.a. | n.a. | |
|  | SP | 11 | 5525 | 5468 | 0.000319 | 0.00091 | 0.00176 | -1.4934 | n.s. | n.a. | n.a. | n.a. | |
|  | WT | 4 | 5525 | 5480 | 0.000517 | 0.002 | 0.00359 | 0.3719 | n.s. | n.a. | n.a. | n.a. | |
| WUS | ALL | 92 | 5843 | 5770 | 0.00003 |  |  | -2.1851 | ** | 0 | 0 | 0 | |
|  | SLC | 64 | 5843 | 5770 | 0.000005 | 0.00113 | 0.00172 | -1.077 | n.s. | n.a. | n.a. | n.a. | |
|  | SL | 17 | 5843 | 5790 | 0.000439 | 0.00093 | 0.00118 | -0.8093 | n.s. | n.a. | n.a. | n.a. | |
|  | SP | 11 | 5843 | 5796 | 0.000929 | 0.00215 | 0.00316 | -0.7691 | n.s. | n.a. | n.a. | n.a. | |
|  | WT | 4 | 5843 | 5798 | 0.009745 | 0.00993 | 0.01018 | -0.8724 | n.s. | n.a. | n.a. | n.a. | |
| ZLL | ALL | 92 | 5282 | 5114 | 0.000004 |  |  | -1.0371 | n.s. | 0 | 0 | 0 | |
|  | SLC | 64 | 5282 | 5114 | 0 | 0.00082 | 0.0011 | n.a. | n.a. | n.a. | n.a. | n.a. | |
|  | SL | 17 | 5282 | 5140 | 0.000383 | 0.00146 | 0.00173 | -2.1407 | ** | n.a. | n.a. | n.a. | |
|  | SP | 11 | 5282 | 5164 | 0.000254 | 0.00108 | 0.00143 | 0.9518 | n.s. | n.a. | n.a. | n.a. | |
|  | WT | 4 | 5282 | 5234 | 0.011623 | 0.01351 | 0.01575 | -0.4036 | n.s. | n.a. | n.a. | n.a. | |
| LC | ALL | 92 | 4997 | 4897 | 0.000009 |  |  | -1.3893 | n.s. | 0 | 0 | 0 | |
|  | SLC | 64 | 4998 | 4897 | 0.000006 | 0.00131 | 0.00189 | -1.077 | n.s. | n.a. | n.a. | n.a. | |
|  | SL | 17 | 4998 | 4960 | 0.000637 | 0.00115 | 0.00134 | -0.6737 | n.s. | n.a. | n.a. | n.a. | |
|  | SP | 11 | 4998 | 4949 | 0.000779 | 0.00108 | 0.00143 | -0.257 | n.s. | n.a. | n.a. | n.a. | |
|  | WT | 4 | 4998 | 4950 | 0.015926 | 0.01705 | 0.01824 | -0.7708 | n.s. | n.a. | n.a. | n.a. | |
| AGO1 | ALL | 92 | 5190 | 5106 | 0.000021 |  |  | -1.7349 | # | 0 | 0 | 0 | |
|  | SLC | 64 | 5190 | 5111 | 0.000018 | 0.00034 | 0.00038 | -1.3153 | n.s. | n.a. | n.a. | n.a. | |
|  | SL | 17 | 5190 | 5160 | 0.000091 | 0.00009 | 0.00009 | -1.8431 | * | n.a. | n.a. | n.a. | |
|  | SP | 11 | 5190 | 5170 | 0.000563 | 0.00119 | 0.00149 | -1.5081 | n.s. | n.a. | n.a. | n.a. | |
|  | WT | 4 | 5190 | 5166 | 0.004839 | 0.00621 | 0.00771 | -0.2582 | n.s. | n.a. | n.a. | n.a. | |
| CLV2 | ALL | 92 | 5057 | 5014 | 0.000004 |  |  | -1.0371 | n.s. | 0 | 0 | 0 | |
|  | SLC | 64 | 5057 | 5014 | 0 | 0.00086 | 0.0015 | n.a. | n.a. | n.a. | n.a. | n.a. | |
|  | SL | 17 | 5057 | 5017 | 0 | 0.00042 | 0.00072 | n.a. | n.a. | n.a. | n.a. | n.a. | |
|  | SP | 11 | 5057 | 5014 | 0.000036 | 0.00135 | 0.0025 | -1.1285 | n.s. | n.a. | n.a. | n.a. | |
|  | WT | 4 | 5057 | 5057 | 0 | 0 | 0 | n.a. | n.a. | n.a. | n.a. | n.a. | |
| NCD | ALL | 92 | 5753 | 5634 | 0.000085 |  |  | -2.4932 | ** | 0 | 0 | 0 | |
|  | SLC | 64 | 5753 | 5639 | 0.00006 | 0.00129 | 0.00159 | -1.9462 | * | n.a. | n.a. | n.a. | |
|  | SL | 17 | 5753 | 5722 | 0.000432 | 0.00033 | 0.00036 | -2.3931 | *** | n.a. | n.a. | n.a. | |
|  | SP | 11 | 5753 | 5700 | 0.001314 | 0.00304 | 0.0041 | -1.245 | n.s. | n.a. | n.a. | n.a. | |
|  | WT | 4 | 5753 | 5638 | 0.026103 | 0.02998 | 0.03381 | -0.7646 | n.s. | n.a. | n.a. | n.a. | |
| REV | ALL | 92 | 7613 | 7466 | 0.000047 |  |  | -2.5158 | *** | 0.0046 | 0.0228 | 0.201754385964912 | |
|  | SLC | 64 | 7613 | 7482 | 0.000063 | 0.00088 | 0.00102 | -2.4553 | ** | n.a. | n.a. | n.a. | |
|  | SL | 17 | 7613 | 7571 | 0.000214 | 0.00033 | 0.00036 | -2.0425 | * | n.a. | n.a. | n.a. | |
|  | SP | 11 | 7613 | 7562 | 0.000548 | 0.00119 | 0.00154 | -0.8448 | n.s. | n.a. | n.a. | n.a. | |
|  | WT | 4 | 7613 | 7466 | 0.010961 | 0.01442 | 0.01815 | -0.4655 | n.s. | n.a. | n.a. | n.a. | |
| FW2.2 | ALL | 92 | 4175 | 4090 | 0.000037 |  |  | -2.1122 | * | 0.0135588028169014 | 0.0238697183098592 | 0.807441613880768 | |
|  | SLC | 64 | 4175 | 4091 | 0.000015 | 0.00155 | 0.00181 | -1.4358 | n.s. | n.a. | n.a. | n.a. | |
|  | SL | 17 | 4175 | 4161 | 0.000283 | 0.00049 | 0.00052 | -2.2053 | ** | n.a. | n.a. | n.a. | |
|  | SP | 11 | 4175 | 4143 | 0.000579 | 0.00197 | 0.00245 | -1.5532 | n.s. | n.a. | n.a. | n.a. | |
|  | WT | 4 | 4175 | 4168 | 0.028111 | 0.02837 | 0.02878 | -0.2009 | n.s. | n.a. | n.a. | n.a. | |
| OVATE | ALL | 92 | 5173 | 5105 | 0.000009 |  |  | -1.3893 | n.s. | 0 | 0 | 0 | |
|  | SLC | 64 | 5173 | 5105 | 0 | 0.00084 | 0.00115 | n.a. | n.a. | n.a. | n.a. | n.a. | |
|  | SL | 17 | 5173 | 5135 | 0.000137 | 0.00055 | 0.00067 | -2.02 | * | n.a. | n.a. | n.a. | |
|  | SP | 11 | 5173 | 5113 | 0.000434 | 0.00264 | 0.00386 | -0.7773 | n.s. | n.a. | n.a. | n.a. | |
|  | WT | 4 | 5173 | 5133 | 0.000292 | 0.00036 | 0.00042 | -0.7545 | n.s. | n.a. | n.a. | n.a. | |
| KOR1 | ALL | 92 | 10542 | 10365 | 0.000012 |  |  | -1.6816 | # | 0 | 0 | 0 | |
|  | SLC | 64 | 10542 | 10373 | 0.000046 | 0.0009 | 0.00118 | -1.705 | # | n.a. | n.a. | n.a. | |
|  | SL | 17 | 10542 | 10462 | 0.00042 | 0.00042 | 0.00042 | 2.3491 | * | n.a. | n.a. | n.a. | |
|  | SP | 11 | 10542 | 10445 | 0.0004 | 0.00139 | 0.00295 | -0.5514 | n.s. | n.a. | n.a. | n.a. | |
|  | WT | 4 | 10542 | 10426 | 0.008888 | 0.0097 | 0.01075 | -0.8043 | n.s. | n.a. | n.a. | n.a. | |
| RBL | ALL | 92 | 9631 | 9414 | 0.000065 |  |  | -2.2146 | ** | 0.00636736842105263 | 0.014 | 0.454812030075188 | |
|  | SLC | 64 | 9631 | 9422 | 0.000074 | 0.00322 | 0.0037 | -1.838 | * | n.a. | n.a. | n.a. | |
|  | SL | 17 | 9631 | 9520 | 0.000745 | 0.00132 | 0.00143 | -2.1309 | ** | n.a. | n.a. | n.a. | |
|  | SP | 11 | 9631 | 9461 | 0.000638 | 0.00368 | 0.00574 | -1.9031 | * | n.a. | n.a. | n.a. | |
|  | WT | 4 | 9631 | 9499 | 0.011036 | 0.01373 | 0.01733 | -0.4595 | n.s. | n.a. | n.a. | n.a. | |
| ANT | ALL | 92 | 3227 | 3135 | 0 |  |  | n.a. | n.a. | 0 | 0 | 0 | |
|  | SLC | 64 | 3227 | 3135 | 0 | 0.00062 | 0.00115 | n.a. | n.a. | n.a. | n.a. | n.a. | |
|  | SL | 17 | 3227 | 3153 | 0.000294 | 0.00067 | 0.0011 | -1.909 | * | n.a. | n.a. | n.a. | |
|  | SP | 11 | 3227 | 3155 | 0.000334 | 0.00094 | 0.0013 | -0.8342 | n.s. | n.a. | n.a. | n.a. | |
|  | WT | 4 | 3227 | 3188 | 0.009933 | 0.01228 | 0.01602 | -0.6635 | n.s. | n.a. | n.a. | n.a. | |
| SUN | ALL | 92 | 4623 | 4550 | 0.000048 |  |  | -2.301 | ** | 0 | 0 | 0 | |
|  | SLC | 64 | 4623 | 4550 | 0.000027 | 0.00156 | 0.00175 | -1.8363 | * | n.a. | n.a. | n.a. | |
|  | SL | 17 | 4623 | 4588 | 0.000654 | 0.00151 | 0.00162 | -1.2472 | n.s. | n.a. | n.a. | n.a. | |
|  | SP | 11 | 4623 | 4564 | 0.000709 | 0.00293 | 0.00418 | -1.8165 | * | n.a. | n.a. | n.a. | |
|  | WT | 4 | 4623 | 4578 | 0.002257 | 0.00319 | 0.00556 | -0.5411 | n.s. | n.a. | n.a. | n.a. | |
| BAM1 | ALL | 92 | 9454 | 9237 | 0.000086 |  |  | -2.5555 | *** | 0.00135714285714286 | 0.0178978835978836 | 0.0774017442749437 | |
|  | SLC | 64 | 9454 | 9251 | 0.000036 | 0.00072 | 0.00084 | -1.7848 | # | n.a. | n.a. | n.a. | |
|  | SL | 17 | 9454 | 9403 | 0.000175 | 0.00019 | 0.00021 | -2.302 | ** | n.a. | n.a. | n.a. | |
|  | SP | 11 | 9454 | 9289 | 0.001045 | 0.00311 | 0.00425 | -1.4379 | n.s. | n.a. | n.a. | n.a. | |
|  | WT | 4 | 9454 | 9293 | 0.015765 | 0.01709 | 0.02034 | -0.7077 | n.s. | n.a. | n.a. | n.a. | |
| TD380 | ALL | 92 | 4254 | 4232 | 0.000068 |  |  | -1.5288 | n.s. | 0.0211136842105263 | 0.0351010526315789 | 0.601595898432633 | |
|  | SLC | 64 | 4254 | 4238 | 0.00008 | 0.00039 | 0.00048 | -1.2905 | n.s. | n.a. | n.a. | n.a. | |
|  | SL | 17 | 4254 | 4244 | 0.00008 | 0.00022 | 0.00024 | -1.0692 | n.s. | n.a. | n.a. | n.a. | |
|  | SP | 11 | 4254 | 4241 | 0.000171 | 0.00044 | 0.00058 | -1.7117 | # | n.a. | n.a. | n.a. | |
|  | WT | 4 | 4254 | 4219 | 0.013036 | 0.01515 | 0.01803 | -0.1197 | n.s. | n.a. | n.a. | n.a. | |
| UFO | ALL | 92 | 4143 | 4099 | 0.000067 |  |  | -1.8975 | * | 0.0152 | 0.0317 | 0.479495268138801 | |
|  | SLC | 64 | 4143 | 4106 | 0.000217 | 0.00163 | 0.00183 | -1.0068 | n.s. | n.a. | n.a. | n.a. | |
|  | SL | 17 | 4143 | 4120 | 0.000518 | 0.00078 | 0.00082 | -1.4983 | n.s. | n.a. | n.a. | n.a. | |
|  | SP | 11 | 4143 | 4123 | 0.000653 | 0.00178 | 0.00229 | -1.4904 | n.s. | n.a. | n.a. | n.a. | |
|  | WT | 4 | 4143 | 4098 | 0.01212 | 0.01404 | 0.0161 | -0.5387 | n.s. | n.a. | n.a. | n.a. | |
| SP | ALL | 92 | 5565 | 5502 | 0.000107 |  |  | -2.6515 | *** | 0 | 0 | 0 | |
|  | SLC | 64 | 5565 | 5513 | 0.000184 | 0.00134 | 0.00143 | -2.3374 | ** | n.a. | n.a. | n.a. | |
|  | SL | 17 | 5565 | 5540 | 0.000297 | 0.00043 | 0.00044 | -2.302 | ** | n.a. | n.a. | n.a. | |
|  | SP | 11 | 5565 | 5526 | 0.000961 | 0.00256 | 0.0034 | -1.008 | n.s. | n.a. | n.a. | n.a. | |
|  | WT | 4 | 5565 | 5319 | 0.014194 | 0.02279 | 0.03605 | -0.8734 | n.s. | n.a. | n.a. | n.a. | |
| AG | ALL | 92 | 4823 | 4755 | 0.000073 |  |  | -1.9126 | * | 0 | 0 | 0 | |
|  | SLC | 64 | 4823 | 4757 | 0.000063 | 0.00055 | 0.00072 | -0.4934 | n.s. | n.a. | n.a. | n.a. | |
|  | SL | 17 | 4823 |  |  | n.a | n.a |  |  | n.a. | n.a. | n.a. | |
|  | SP | 17 | 4823 | 4801 | 0.000074 |  |  | -1.7057 | # | n.a. | n.a. | n.a. | |
|  | WT | 4 | 4823 | 4763 | 0.014452 | 0.01653 | 0.02032 | -0.7554 | n.s. | n.a. | n.a. | n.a. | |

| **Additional file 6: List of Arabidopsis candidate proteins (TAIR 10). TBLASTn on tomato genome sequence (v2.40) output** |
| --- |
|  |
| Query: AT5G19280.1 \| Symbols: KAPP, RAG1 \| KAPP (KINASE ASSOCIATED PROTEIN PHOSPHATASE); phosphoprotein phosphatase/ protein kinase binding / protein serine/threonine phosphatase \| chr5:6488450-6493182 FORWARD |
| Sbjct: Solyc01g079720.2.1 genomic_reference:SL2.40ch01 gene_region:71336146-71351250 transcript_region:SL2.40ch01:71336146..71351250- go_terms:GO:0004721,GO:0019901 functional_description:"Kinase-associated protein phosphatase 1 (AHRD V1 **** A6N8J3_SOLPE); contains Interpro domain(s) IPR015655 Protein phosphatase 2C " |
| tblastn // Sbjct : 362 2023 2285 +2 // Query : 27 580 581 0 // S=623 E=1e-178 I=313/565 (55%) Pos=418/565 (73%) |
|  |
| Query: AT2G45190.1 \| Symbols: AFO, FIL, YAB1 \| AFO (ABNORMAL FLORAL ORGANS); protein binding / transcription factor/ transcription regulator \| chr2:18628450-18630552 REVERSE |
| Sbjct: Solyc01g091010.2.1 genomic_reference:SL2.40ch01 gene_region:76475369-76478983 transcript_region:SL2.40ch01:76475369..76478983- go_terms:GO:0005515,GO:0003700 functional_description:"YABBY-like transcription factor CRABS CLAW-like protein (AHRD V1 **-* Q6SRZ7_ANTMA); contains Interpro domain(s) IPR006780 YABBY protein " |
| tblastn // Sbjct : 252 836 1077 +3 // Query : 22 229 229 0 // S=250 E=5e-67 I=137/210 (65%) Pos=160/210 (76%) |
|  |
| Query: AT2G34710.1 \| Symbols: PHB, ATHB14, ATHB-14, PHB-1D \| PHB (PHABULOSA); DNA binding / transcription factor \| chr2:14639548-14643993 REVERSE |
| Sbjct: Solyc02g024070.2.1 genomic_reference:SL2.40ch02 gene_region:15627267-15633567 transcript_region:SL2.40ch02:15627267..15633567+ go_terms:GO:0005515,GO:0003677 functional_description:"Class III homeodomain-leucine zipper (AHRD V1 ***- Q1WD30_GINBI); contains Interpro domain(s) IPR013978 MEKHLA " |
| tblastn // Sbjct : 282 2783 2974 +3 // Query : 21 852 852 0 // S=1340 E=0.0 I=657/839 (78%) Pos=728/839 (86%) |
|  |
| Query: AT4G18960.1 \| Symbols: AG \| AG (AGAMOUS); DNA binding / transcription factor \| chr4:10383917-10388272 FORWARD |
| Sbjct: Solyc02g071730.2.1 genomic_reference:SL2.40ch02 gene_region:35671866-35677320 transcript_region:SL2.40ch02:35671866..35677320- go_terms:GO:0005515,GO:0003700 functional_description:"MADS-box transcription factor AGAMOUS (AHRD V1 **-* Q8GTY3_HELAN); contains Interpro domain(s) IPR002100 Transcription factor, MADS-box IPR002487 Transcription factor, K-box " |
| tblastn // Sbjct : 126 863 993 +3 // Query : 3 252 252 0 // S=329 E=1e-90 I=170/254 (66%) Pos=197/254 (77%) |
|  |
| Query: AT2G17950.1 \| Symbols: WUS, PGA6, WUS1 \| WUS (WUSCHEL); DNA binding / protein binding / transcription factor/ transcription regulator \| chr2:7809100-7810671 REVERSE |
| Sbjct: Solyc02g083950.2.1 genomic_reference:SL2.40ch02 gene_region:41768119-41769544 transcript_region:SL2.40ch02:41768119..41769544- go_terms:GO:0006355 functional_description:"WUSCHEL-related homeobox-containing protein 4 (AHRD V1 *-*- C0LAL8_9MAGN); contains Interpro domain(s) IPR001356 Homeobox " |
| tblastn // Sbjct : 92 307 1006 +2 // Query : 30 101 292 0 // S=132 E=2e-31 I=56/72 (77%) Pos=69/72 (95%) |
|  |
| Query: AT1G48410.1 \| Symbols: AGO1 \| AGO1 (ARGONAUTE 1); endoribonuclease/ miRNA binding / protein binding / siRNA binding \| chr1:17886285-17891892 REVERSE |
| Sbjct: Solyc06g072300.2.1 genomic_reference:SL2.40ch06 gene_region:40960724-40969540 transcript_region:SL2.40ch06:40960724..40969540- go_terms:GO:0019899 functional_description:"ARGONAUTE 1 (AHRD V1 ***- D6RUV9_TOBAC); contains Interpro domain(s) IPR003165 Stem cell self-renewal protein Piwi " |
| tblastn // Sbjct : 2106 4931 5143 +3 // Query : 115 1048 1048 0 // S=1578 E=0.0 I=766/943 (81%) Pos=824/943 (87%) |
|  |
| Query: AT1G15750.1 \| Symbols: WSIP1, TPL \| TPL (TOPLESS); protein binding / protein homodimerization/ transcription repressor \| chr1:5415086-5420359 REVERSE |
| Sbjct: Solyc03g117360.2.1 genomic_reference:SL2.40ch03 gene_region:60573693-60584026 transcript_region:SL2.40ch03:60573693..60584026+ go_terms:GO:0042803,GO:0016564 functional_description:"WD-40 repeat protein-like (Fragment) (AHRD V1 *--- Q1HIU4_9ROSI); contains Interpro domain(s) IPR017986 WD40 repeat, region " |
| tblastn // Sbjct : 1 3390 3774 +1 // Query : 1 1131 1131 0 // S=1865 E=0.0 I=898/1134 (79%) Pos=975/1134 (85%) |
|  |
| Query: AT1G52150.1 \| Symbols: ATHB-15, ATHB15, CNA, ICU4 \| ATHB-15; DNA binding / transcription factor \| chr1:19409913-19413961 REVERSE |
| Sbjct: Solyc03g120910.2.1 genomic_reference:SL2.40ch03 gene_region:63195847-63202781 transcript_region:SL2.40ch03:63195847..63202781+ go_terms:GO:0003700 functional_description:"Class III homeodomain-leucine zipper (AHRD V1 ***- Q1WD30_GINBI); contains Interpro domain(s) IPR013978 MEKHLA " |
| tblastn // Sbjct : 308 2809 2982 +2 // Query : 4 836 836 0 // S=1466 E=0.0 I=707/838 (84%) Pos=764/838 (91%) |
|  |
| Query: AT1G55580.1 \| Symbols: LAS, SCL18 \| LAS (Lateral Suppressor); transcription factor \| chr1:20764106-20765443 FORWARD |
| Sbjct: Solyc07g066250.1.1 evidence_code:10F0H1E0IEG genomic_reference:SL2.40ch07 gene_region:64958148-64959434 transcript_region:SL2.40ch07:64958148..64959434+ go_terms:GO:0003700 functional_description:"GRAS family transcription factor (Fragment) (AHRD V1 **-* B1Q3B1_BRACM); contains Interpro domain(s) IPR005202 GRAS transcription factor " |
| tblastn // Sbjct : 145 1284 1287 +1 // Query : 41 445 445 0 // S=382 E=1e-106 I=205/405 (50%) Pos=258/405 (63%) |
|  |
| Query: AT1G62360.1 \| Symbols: STM, BUM1, SHL, WAM1, BUM, WAM \| STM (SHOOT MERISTEMLESS); transcription factor \| chr1:23058796-23061722 REVERSE |
| Sbjct: Solyc02g081120.2.1 genomic_reference:SL2.40ch02 gene_region:39767063-39773953 transcript_region:SL2.40ch02:39767063..39773953+ go_terms:GO:0005515 functional_description:"Knotted-1-like homeobox protein H1 (AHRD V1 ***- Q8GUS6_TOBAC); contains Interpro domain(s) IPR005541 KNOX2 " |
| tblastn // Sbjct : 646 1410 1613 +1 // Query : 126 381 382 0 // S=370 E=1e-103 I=195/258 (75%) Pos=206/258 (79%) |
|  |
| Query: AT1G65380.1 \| Symbols: CLV2, AtRLP10 \| CLV2 (clavata 2); protein binding / receptor signaling protein \| chr1:24286943-24289105 FORWARD |
| Sbjct: Solyc04g056640.1.1 evidence_code:10F0H1E1IEG genomic_reference:SL2.40ch04 gene_region:53775339-53777579 transcript_region:SL2.40ch04:53775339..53777579+ go_terms:GO:0004675 functional_description:"LRR receptor-like serine/threonine-protein kinase, RLP" |
| tblastn // Sbjct : 163 2226 2241 +1 // Query : 33 719 720 0 // S=707 E=0.0 I=385/695 (55%) Pos=485/695 (69%) |
|  |
| Query: AT4G37750.1 \| Symbols: ANT, DRG, CKC, CKC1 \| ANT (AINTEGUMENTA); DNA binding / transcription factor \| chr4:17739782-17742189 FORWARD |
| Sbjct: Solyc02g092050.2.1 genomic_reference:SL2.40ch02 gene_region:47836352-47839882 transcript_region:SL2.40ch02:47836352..47839882- go_terms:GO:0003677 functional_description:"AP2-like ethylene-responsive transcription factor At1g16060 (AHRD V1 *-*- AP2L1_ARATH); contains Interpro domain(s) IPR001471 Pathogenesis-related transcriptional factor and ERF, DNA-binding " |
| tblastn // Sbjct : 337 1737 2275 +1 // Query : 66 491 555 0 // S=421 E=1e-118 I=246/496 (49%) Pos=286/496 (57%) |
|  |
| Query: AT4G24190.1 \| Symbols: SHD, HSP90.7 \| SHD (SHEPHERD); ATP binding / unfolded protein binding \| chr4:12551902-12555851 REVERSE |
| Sbjct: Solyc04g081570.2.1 genomic_reference:SL2.40ch04 gene_region:63110553-63116088 transcript_region:SL2.40ch04:63110553..63116088- go_terms:GO:0042623,GO:0050750 functional_description:"Chaperone protein htpG (AHRD V1 **-- HTPG_MYCA1); contains Interpro domain(s) IPR015566 Molecular chaperone, heat shock protein, endoplasmin " |
| tblastn // Sbjct : 1117 2421 2792 +1 // Query : 340 775 823 0 // S=773 E=0.0 I=372/436 (85%) Pos=410/436 (94%) |
|  |
| Query: AT1G75820.1 \| Symbols: CLV1, FAS3, FLO5 \| CLV1 (CLAVATA 1); ATP binding / kinase/ protein serine/threonine kinase/ receptor signaling protein serine/threonine kinase \| chr1:28463631-28466652 REVERSE |
| Sbjct: Solyc04g081590.2.1 genomic_reference:SL2.40ch04 gene_region:63126375-63130644 transcript_region:SL2.40ch04:63126375..63130644- go_terms:GO:0004675 functional_description:"Receptor like kinase, RLK" |
| tblastn // Sbjct : 98 2965 3304 +2 // Query : 24 976 980 0 // S=1246 E=0.0 I=607/957 (63%) Pos=743/957 (77%) |
|  |
| Query: AT4G28190.1 \| Symbols: ULT1, ULT \| ULT1 (ULTRAPETALA1); DNA binding \| chr4:13985753-13987050 FORWARD |
| Sbjct: Solyc07g054450.2.1 genomic_reference:SL2.40ch07 gene_region:60092916-60096795 transcript_region:SL2.40ch07:60092916..60096795+ go_terms:GO:0005634 functional_description:"Transcription factor (Fragment) (AHRD V1 ***- D6MKF6_9ASPA); contains Interpro domain(s) IPR000770 SAND " |
| tblastn // Sbjct : 209 874 1103 +2 // Query : 12 237 237 0 // S=336 E=7e-93 I=157/226 (69%) Pos=189/226 (83%) |
|  |
| Query: AT1G30490.1 \| Symbols: PHV, ATHB9 \| PHV (PHAVOLUTA); DNA binding / protein binding / transcription factor \| chr1:10796328-10800744 REVERSE |
| Sbjct: Solyc02g024070.2.1 genomic_reference:SL2.40ch02 gene_region:15627267-15633567 transcript_region:SL2.40ch02:15627267..15633567+ go_terms:GO:0005515,GO:0003677 functional_description:"Class III homeodomain-leucine zipper (AHRD V1 ***- Q1WD30_GINBI); contains Interpro domain(s) IPR013978 MEKHLA " |
| tblastn // Sbjct : 282 2783 2974 +3 // Query : 17 841 841 0 // S=1304 E=0.0 I=640/841 (76%) Pos=719/841 (85%) |
|  |
| Query: AT5G43810.1 \| Symbols: ZLL, PNH, AGO10 \| ZLL (ZWILLE); translation initiation factor \| chr5:17611939-17616562 FORWARD |
| Sbjct: Solyc09g082830.2.1 genomic_reference:SL2.40ch09 gene_region:63888767-63895211 transcript_region:SL2.40ch09:63888767..63895211- go_terms:GO:0019899 functional_description:"ARGONAUTE 1 (AHRD V1 ***- D6RUV9_TOBAC); contains Interpro domain(s) IPR003165 Stem cell self-renewal protein Piwi " |
| tblastn // Sbjct : 474 3050 3240 +3 // Query : 128 988 988 0 // S=1578 E=0.0 I=753/862 (87%) Pos=810/862 (93%) |
|  |
| Query: AT5G60690.1 \| Symbols: REV, IFL, IFL1 \| REV (REVOLUTA); DNA binding / lipid binding / transcription factor \| chr5:24397734-24401933 FORWARD |
| Sbjct: Solyc11g069470.1.1 evidence_code:10F1H1E1IEG genomic_reference:SL2.40ch11 gene_region:51150759-51156271 transcript_region:SL2.40ch11:51150759..51156271+ go_terms:GO:0006355,GO:0045449 functional_description:"Class III homeodomain-leucine zipper (AHRD V1 ***- Q1WD30_GINBI); contains Interpro domain(s) IPR013978 MEKHLA " |
| tblastn // Sbjct : 1 2523 2526 +1 // Query : 1 842 842 0 // S=1377 E=0.0 I=659/845 (77%) Pos=729/845 (86%) |
|  |
| Query: AT5G49720.1 |
| Sbjct: Solyc01g102580.2.1 genomic_reference:SL2.40ch01 gene_region:83117822-83121772 transcript_region:SL2.40ch01:83117822..83121772- go_terms:GO:0008810 functional_description:"Endo-1 4-beta-glucanase (AHRD V1 ***- O04890_SOLLC); contains Interpro domain(s) IPR008928 Six-hairpin glycosidase-like IPR018221 Glycoside hydrolase, family 9, active site IPR001701 Glycoside hydrolase, family 9 " |
| tblastn // Sbjct : 323 2125 2515 +2 // Query : 1 605 621 0 // S=870 E=0.0 I=423/605 (69%) Pos=461/605 (76%) |
|  |
| Query: AT1G30950.1 |
| Sbjct: Solyc02g081670.1.1 evidence_code:10F1H1E1IEG genomic_reference:SL2.40ch02 gene_region:40120235-40121602 transcript_region:SL2.40ch02:40120235..40121602+ go_terms:GO:0008134,GO:0004842 functional_description:"Fimbriata (Fragment) (AHRD V1 **-- Q6QVW9_MIMLE); contains Interpro domain(s) IPR001810 Cyclin-like F-box " |
| tblastn // Sbjct : 127 1353 1368 +1 // Query : 41 438 442 0 // S=492 E=1e-139 I=243/410 (59%) Pos=298/410 (72%) |
|  |
| Query: AT5G66240.2 |
| Sbjct: Solyc02g083940.2.1 genomic_reference:SL2.40ch02 gene_region:41756601-41762285 transcript_region:SL2.40ch02:41756601..41762285+ go_terms:GO:0042800 functional_description:"WD repeat protein-like (AHRD V1 **-- Q8RXD8_ARATH); contains Interpro domain(s) IPR020472 G-protein beta WD-40 repeat, region " |
| tblastn // Sbjct : 16 996 1020 +1 // Query : 5 331 331 0 // S=544 E=1e-155 I=263/327 (80%) Pos=292/327 (89%) |
|  |
| Query: AT5G66750.1 |
| Sbjct: Solyc02g062780.2.1 genomic_reference:SL2.40ch02 gene_region:29133439-29143895 transcript_region:SL2.40ch02:29133439..29143895- go_terms:GO:0004003 functional_description:"Chromodomain-helicase-DNA- binding protein 6 (AHRD V1 **-* CHD6_HUMAN); contains Interpro domain(s) IPR000330 SNF2-related " |
| tblastn // Sbjct : 97 2313 2547 +1 // Query : 27 764 764 0 // S=1028 E=0.0 I=509/742 (68%) Pos=592/742 (79%) |
|  |
| Query: AT2G18500.1 |
| Sbjct: Solyc03g034100.2.1 genomic_reference:SL2.40ch03 gene_region:10070572-10072340 transcript_region:SL2.40ch03:10070572..10072340+ functional_description:"Plant-specific domain TIGR01568 family protein (AHRD V1 *--- B6U561_MAIZE); contains Interpro domain(s) IPR006458 Protein of unknown function DUF623, plant " |
| tblastn // Sbjct : 942 1190 1344 +3 // Query : 206 290 315 0 // S=99.0 E=4e-21 I=52/85 (61%) Pos=58/85 (68%) |
|  |
| Query: AT1G14870.1 |
| Sbjct: Solyc01g005470.2.1 genomic_reference:SL2.40ch01 gene_region:322628-324932 transcript_region:SL2.40ch01:322628..324932- functional_description:"Cell number regulator 10 (AHRD V1 **-- D9HP26_MAIZE); contains Interpro domain(s) IPR006461 Protein of unknown function Cys-rich " |
| tblastn // Sbjct : 163 573 750 +1 // Query : 13 152 152 0 // S=196 E=5e-51 I=94/140 (67%) Pos=109/140 (77%) |
|  |
| Query: AT5G03960.1 |
| Sbjct: Solyc04g016480.2.1 genomic_reference:SL2.40ch04 gene_region:7305326-7308804 transcript_region:SL2.40ch04:7305326..7308804- go_terms:GO:0005516 functional_description:"Calmodulin binding protein (AHRD V1 *--* B4FU94_MAIZE); contains Interpro domain(s) IPR000048 IQ calmodulin-binding region " |
| tblastn // Sbjct : 123 1211 1551 +3 // Query : 1 358 403 0 // S=107 E=2e-23 I=99/371 (26%) Pos=164/371 (44%) |
|  |
| Query: AT2G28290.1 |
| Sbjct: Solyc11g062010.1.1 evidence_code:10F0H1E0IEG genomic_reference:SL2.40ch11 gene_region:45838303-45866988 transcript_region:SL2.40ch11:45838303..45866988+ go_terms:GO:0005524 functional_description:"Chromodomain-helicase-DNA-binding protein 1 (AHRD V1 *--- CHD1_DROME); contains Interpro domain(s) IPR000330 SNF2-related " |
| tblastn // Sbjct : 1291 4803 8004 +1 // Query : 339 1485 3574 0 // S=1377 E=0.0 I=708/1191 (59%) Pos=828/1191 (69%) |
|  |
| Query: AT5G65700.1 |
| Sbjct: Solyc02g091840.2.1 genomic_reference:SL2.40ch02 gene_region:47648877-47652967 transcript_region:SL2.40ch02:47648877..47652967+ go_terms:GO:0004675 functional_description:"Receptor like kinase, RLK" |
| tblastn // Sbjct : 265 3222 3516 +1 // Query : 12 999 1003 0 // S=1538 E=0.0 I=751/991 (75%) Pos=851/991 (85%) |
|  |
| Query: AT5G03840.1 |
| Sbjct: Solyc06g074350.2.1 genomic_reference:SL2.40ch06 gene_region:42361623-42363883 transcript_region:SL2.40ch06:42361623..42363883+ go_terms:GO:0005515,GO:0008429 functional_description:"self-pruning" |
| tblastn // Sbjct : 82 606 859 +1 // Query : 4 177 177 0 // S=264 E=2e-71 I=121/175 (69%) Pos=151/175 (86%) |
|  |
| Query: AT3G55510.1 |
| Sbjct: Solyc05g023710.2.1 genomic_reference:SL2.40ch05 gene_region:29024158-29035186 transcript_region:SL2.40ch05:29024158..29035186+ functional_description:"Nucleolar complex protein 2 homolog (AHRD V1 ***- C0H9R3_SALSA); contains Interpro domain(s) IPR005343 Uncharacterised protein family UPF0120 " |
| tblastn // Sbjct : 349 1800 2188 +1 // Query : 107 593 594 0 // S=461 E=1e-130 I=241/493 (48%) Pos=337/493 (68%) |

| **Additional file 7: Phenotypic data Information on 96 accessions used in the association study and diversity analysis** | | | | |  |  |
| --- | --- | --- | --- | --- | --- | --- |
| Accession Number | Accession Name | species | mature fruit color | Traits ^a^ | |  |
|  |  |  |  | FW (g) | LCN | FSI |
| CR001 | Cervil | *S.l.cerasiforme* | red | 5.796018 | 2.183998 | 0.8752 |
| CR002 | Levovil | *S.lycopersicum* | red | 109.131417 | 3.910619 | 0.7959 |
| CR003 | Ferum | *S.lycopersicum* | red | 109.664431 | 2.23837 | 0.8806 |
| CR004 | M-82 | *S.lycopersicum* | red | 62.601018 | 2.452516 | 1.0552 |
| CR014 | Clémentine | *S.l.cerasiforme* | red | 5.404351 | 2.252516 | 0.9989 |
| CR020 | San Marzano | *S.lycopersicum* | red | 70.004351 | 2.245109 | 1.2742 |
| CR028 | Plovdiv XXIVa | *S.l.cerasiforme* | red | 41.579351 | 2.050665 | 1.094 |
| CR031 | Microtom | *S.lycopersicum* | red | 6.914351 | 3.167331 | 0.8761 |
| CR032 | Moneymaker | *S.lycopersicum* | red | 99.536018 | 2.491934 | 0.8409 |
| CR056 | Wva 700 | *S.l.cerasiforme* | red | 4.254351 | 2.100665 | 0.9684 |
| CR058 | Wva 106 | *S.l.cerasiforme* | red | 9.687685 | 2.717331 | 0.8483 |
| CR062 | LA 1478 | *S.pimpinellifolium* | red | 2.054351 | 2.300665 | 0.9736 |
| CR068 | N° 108 Red Currant | *S.pimpinellifolium* | red | 2.004351 | 1.983998 | 1.0114 |
| CR070 | N° 2909 Lycopersicon sp. | *S.l.cerasiforme* | red | 5.121018 | 2.050665 | 1.1414 |
| CR072 | N° 2921 Lyc. Pimpinellifolium | *S.pimpinellifolium* | red | 2.154351 | 2.017331 | 0.9724 |
| CR075 | N° 4156 Blumen Strauss | *S.pimpinellifolium* | red | 1.662685 | 1.933998 | 1.2388 |
| CR076 | N° 135 Green Gage | *S.l.cerasiforme* | red | 39.904351 | 2.133998 | 0.8765 |
| CR077 | N°1565 | *S.l.cerasiforme* | red | 10.187685 | 2.283998 | 0.8364 |
| CR078 | N° 2759 Enano | *S.l.cerasiforme* | red | 34.837685 | 3.333998 | 0.8565 |
| CR079 | N° 933 | *S.l.cerasiforme* | red | 33.637685 | 3.817331 | 0.8292 |
| CR093 | N° 2257 Dikorastushii... | *S.l.cerasiforme* | red | 23.546018 | 3.583998 | 0.7908 |
| CR094 | N° 1011 Srednei Velichiny | *S.lycopersicum* | red | 23.297685 | 2.978442 | 0.9184 |
| CR097 | N° 347 Yablochnyi | *S.l.cerasiforme* | red | 30.487685 | 2.350665 | 0.8131 |
| CR098 | N° 795 Pescio | *S.l.cerasiforme* | red | 22.107685 | 3.817331 | 0.8917 |
| CR101 | N° 884 Alagabotskii | *S.l.cerasiforme* | red | 24.487685 | 3.350665 | 0.7787 |
| CR102 | N° 739 | *S.l.cerasiforme* | red | 54.129351 | 7.521498 | 0.6122 |
| CR106 | LA 1025 | *S.l.cerasiforme* | red | 15.079351 | 2.100665 | 0.9139 |
| CR108 | LA 1231 | *S.l.cerasiforme* | red | 4.987685 | 2.183998 | 0.9525 |
| CR110 | LA 1307 | *S.l.cerasiforme* | red | 14.746018 | 2.152516 | 0.9598 |
| CR117 | LA 1388 | *S.l.cerasiforme* | red | 16.887685 | 4.046961 | 0.7936 |
| CR118 | LA 1420 | *S.l.cerasiforme* | red | 39.682685 | 5.132146 | 0.8141 |
| CR122 | LA 1456 | *S.l.cerasiforme* | red | 4.964351 | 2.000665 | 1.0645 |
| CR123 | LA 1461 | *S.l.cerasiforme* | red | 3.934351 | 2.017331 | 0.9056 |
| CR124 | LA 1464 | *S.l.cerasiforme* | red | 3.249351 | 2.017331 | 0.9402 |
| CR125 | LA 1482 | *S.l.cerasiforme* | red | 9.659351 | 2.300665 | 0.9018 |
| CR129 | LA 0147 | *S.lycopersicum* | red | 116.771018 | 3.939553 | 0.7893 |
| CR130 | LA 0172 | *S.l.cerasiforme* | red | 37.681018 | 3.483998 | 0.76 |
| CR133 | LA 0409 | *S.lycopersicum* | red | 116.722685 | 15.479236 | 0.7208 |
| CR134 | LA 0466 | *S.lycopersicum* | red | 208.894351 | 12.711776 | 0.7041 |
| CR136 | LA 0473 | *S.lycopersicum* | red | 49.887685 | 9.533998 | 0.6327 |
| CR145 | LA 1543 | *S.l.cerasiforme* | red | 11.267685 | 2.150665 | 1.3821 |
| CR149 | LA 2095 | *S.l.cerasiforme* | red | 26.904351 | 3.598813 | N/A |
| CR150 | LA 2131 | *S.l.cerasiforme* | red | 40.357685 | 4.511776 | 0.8518 |
| CR152 | LA 2307 | *S.l.cerasiforme* | red | 25.999351 | 3.328442 | 0.7592 |
| CR153 | LA 2308 | *S.l.cerasiforme* | red | 27.699351 | 2.915479 | 0.8699 |
| CR155 | LA 2402 | *S.l.cerasiforme* | red | 6.771018 | 2.233998 | 0.7049 |
| CR156 | LA 2619 | *S.l.cerasiforme* | red | 13.771018 | 4.133998 | 0.7519 |
| CR158 | LA 2675 | *S.l.cerasiforme* | red | 4.987685 | 2.000665 | 0.9392 |
| CR159 | LA 2688 | *S.l.cerasiforme* | red | 4.337685 | 2.000665 | 0.9958 |
| CR163 | LA 0400 | *S.pimpinellifolium* | red | 2.104351 | 2.083998 | 0.9062 |
| CR164 | LA 0411 | *S.pimpinellifolium* | red | 3.137685 | 2.150665 | 0.9176 |
| CR169 | LA 1371 | *S.pimpinellifolium* | red | 2.304351 | 2.033998 | 0.9639 |
| CR173 | LA 1547 | *S.pimpinellifolium* | red | 3.421018 | 2.000665 | 0.9174 |
| CR186 | LA 1689 | *S.pimpinellifolium* | red | 2.204351 | 2.133998 | 0.874 |
| CR199 | tomate Richter's | *S.l.cerasiforme* | red | 3.821018 | 2.067331 | 0.9472 |
| CR202 | CGN 18399 | *S.l.cerasiforme* | red | 6.454351 | 2.083998 | 0.91 |
| CR203 | LA 1589 | *S.pimpinellifolium* | red | 2.404351 | 2.083998 | 0.8996 |
| CR205 | L. pimpinellifolium atypique, site 10 (F300045) | *S.l.cerasiforme* | red | 10.421018 | 2.150665 | 1.1253 |
| CR234 | Atom | *S.l.cerasiforme* | red | 26.496018 | 2.533998 | 0.9738 |
| CR236 | PI 365923 | *S.l.cerasiforme* | red | 15.321018 | 2.083998 | 0.8764 |
| CR238 | PI 129088 | *S.l.cerasiforme* | red | 12.334351 | 3.154831 | 0.9843 |
| CR240 | L 285 | *S.l.cerasiforme* | red | 15.962685 | 2.158998 | 0.9402 |
| CR244 | Yellow Pear | *S.l.cerasiforme* | red | 19.054351 | 2.350665 | 1.5405 |
| CR249 | Cherry Gold | *S.l.cerasiforme* | red | 7.526018 | 2.55622 | 0.884 |
| CR250 | Cherry VFNT | *S.l.cerasiforme* | red | 21.601018 | 2.000665 | 0.8671 |
| CR252 | Droplet | *S.l.cerasiforme* | red | 16.712685 | 2.217331 | 1.4354 |
| CR253 | Monplaisir | *S.l.cerasiforme* | red | 22.487685 | 2.217331 | 0.8812 |
| CR254 | Farthest North | *S.l.cerasiforme* | red | 8.912685 | 3.133998 | 0.9394 |
| CR256 | Minibel | *S.l.cerasiforme* | red | 19.454351 | 4.167331 | 0.7867 |
| CR258 | Ohmiya Suncherry | *S.l.cerasiforme* | red | 13.821018 | 2.100665 | 0.8732 |
| CR267 | Tiny tim | *S.l.cerasiforme* | red | 11.079351 | 2.800665 | 0.8867 |
| CR271 | Celsior | *S.l.cerasiforme* | red | 12.121018 | 2.017331 | 1.6728 |
| CR273 | Orange Cocktail | *S.lycopersicum* | red | 60.707685 | 4.072093 | 0.9093 |
| CR274 | Marpha n°2 | *S.l.cerasiforme* | red | 8.537685 | 3.300665 | 0.7901 |
| CR275 | Cerise Ildi | *S.l.cerasiforme* | red | 7.654351 | 2.600665 | 1.1476 |
| CR279 | Cerise Orange d'Uzès | *S.l.cerasiforme* | red | 13.821018 | 2.267331 | 0.9619 |
| CR280 | Cerise du sud ouest n° 2 | *S.l.cerasiforme* | red | 10.221018 | 2.150665 | 0.8816 |
| CR284 | cerise rose | *S.l.cerasiforme* | red | 10.454351 | 2.800665 | 0.9276 |
| CR287 | Cisterno | *S.l.cerasiforme* | red | 21.554351 | 2.350665 | 0.8742 |
| CR288 | Criollo | *S.l.cerasiforme* | red | 26.109351 | 3.672887 | 0.7505 |
| CR291 | Pyriforme | *S.l.cerasiforme* | red | 10.037685 | 2.033998 | 1.3245 |
| CR292 | 8 bis | *S.l.cerasiforme* | red | 20.654351 | 2.217331 | 0.9473 |
| CR293 | Costa Rica | *S.l.cerasiforme* | red | 15.871018 | 3.167331 | 0.8772 |
| CR294 | Phyra | *S.l.cerasiforme* | red | 5.254351 | 2.217331 | 1.1248 |
| CR296 | Poire jaune | *S.l.cerasiforme* | red | 16.942685 | 2.229831 | 1.5153 |
| CR317 | Heinz 1706 | *S.lycopersicum* | red | 43.704351 | 2.500665 | 1.3732 |
| CR321 | Edkawy | *S.lycopersicum* | red | 224.301018 | 11.182411 | 0.6418 |
| CR341 | Cra 66 | *S.lycopersicum* | red | 40.454351 | 5.183998 | 0.7926 |
| CR354 | Stupicke Polni Rane | *S.lycopersicum* | red | 61.211018 | 4.15622 | 0.8398 |
| CR359 | Muchamiel | *S.lycopersicum* | red | 172.942685 | 5.539553 | 0.8088 |
| CR206 | PI247087 | *S.habrochaites* | green | N/A | N/A | N/A |
| CR207 | LA716 | *S.pennellii* | green | N/A | N/A | N/A |
| CR228 | LA1401 | *S.chesmaniae* | orange | N/A | N/A | N/A |
| CR00X | X | *S.chmielewskii* | green | N/A | N/A | N/A |
| ^a^ Values for fruit weight (FW), locule number (LCN) are adjusted mean from two years of experiment | | | |  |  |  |

| **Additional file 8:** Primer list for re-sequenced candidates (v.2.40) | | |
| --- | --- | --- |
| Fragment_ID | Primer Seq F | Primer R Seq |
| CLV1 | ACAACAGGGCAAAGACAAATCAA | CAGCTTCCAATTCACCACAAAAC |
| CNA | TGGCTGCTAAACTCCTTCTACCC | TGTTGGCTGCTTCAACTTTCTTC |
| FIL-YAB1 | GGGATCAGAGTCAAAACCCATCT | CAAACGTCCCTTTCTCACTCCTT |
| KAPP | GATTTCATATCGCACGACCAAAC | AGTCCCAGGCAAACCAAAATAGA |
| LAS | CTTTTCTACATTGCCTCCAACCC | TTGTAATTTCCCACTCAAGCCAA |
| PHB | GGTGACACAGGGAAGGATTTAGG | TGGGGTTGGGAGAGTATTGTTTT |
| PHV | CACCAGAGTCCGATTTCCTTCTT | TTTATTCTCTCGCCTCTCGCTCT |
| SHD | ATTTGTGTAAATCAGCCAGCCAC | GAAGAACCAATTAAAATCCGCCA |
| STM | ATTCACACCTTTGTTGTGCGTCT | CCTTACCTCCCCAAATAGGCTTC |
| SYD | TAACTGCATCCTGCCCTACAAAA | TGTGCATCACCTACTTCACCAGA |
| TPL | TGTCGTGCACATTCATCTATCATCG | TCTTGGTCCTTCATCTATTCCAGGTA |
| ULT1 | ACGAGTCCATTCACCTTTTGCTT | TACCCCTCCACCTACTTGCCTAC |
| WUS | GATTTGGACTTTTGGGTTTGTCC | TCAATTTCAGTCCTTCTCTCCCA |
| ZLL-PNH-AGO10 | TTCCACTCCTATCAATGCTGCTC | AAAAACCCCAAGACCACTCAAAA |
| LC | CCGGTTCTTCTGAGCTTTCATTT | CGTGCCTTGAACATTTCTGTTTT |
| AGO1 | TGCTGTGTCAACTAGTTGCTTGTTCAGT | CAGGCTATGAAGAACACCGGAATGCTC |
| CLV2 | CGATAAGCGCAACATGACAAATGATTCCGA | TGTACTCAGTCTTACTCGCAGCGTTGA |
| *CONTROL* | CGTATACAGTTCACCTCTCTCCCACTGTA | GCTTCCCTTCATTAATTATACACCACTCTCTAT |
| REV | GGTACCTCTCCGTCTTAATTTACGTAACA | ACAATCATCTAAACAAGGTGTGACCATGC |
| FW2.2/ATPCR2 | GTGGCCAAGATCCAACCATTTCATCAATTG | GTTATCTTCTTCAACTCAACTGCTCCTTGTTCG |
| OVATE/ATOFP7 | CTCAGAAAGAGTATTGCAGGTTACTCTG | GACTTATGAGTTGTATTGGCCTGACG |
| KOR1 | GACGCCATAACTTCAACGAGTTCTTA | AACATGATCTCTATGATATGCATTGGC |
| RBL | CCACATAGGCTTATTGATAGAATCATTGC | GCATCTAATATTGGGAGATGCAACATG |
| ANT | GTGCCACATCAACTCATCAAGACATGA | CAGTATGCTGAACAGCATGCTATTGAC |
| SUN | CTTAGGATTCGTGCTATTGCTAGGTA | CTTCTGCAACACTATCGTGCGA |
| BAM1 | CATGGCCATACCAACACTACTAGGTC | GACTATCACTTCATGAGGTGCTTCAATGA |
| TD380 /DDM1 | TGCTTCTTCCCTGTTTCTTCTTTCT | AAGGTGACAACAAAACTAGCCTTCC |
| UFO | ATACCAAACCAAACGGAGGATAAGA | GCCTTCGGGAGTAAGATAACTGAAA |
| SP/TFL1 | AAGCTCCTTGTATGGGAAAAGATTG | GTTGTCGTACAGTTGATTAGACGGG |
| AG | GTGATCTAACCAGAGAGATCTCACCA | CTGGCATCAAGTTCATCTGCTGA |
