# Supplementary material for: Genes involved in floral meristem in tomato exhibit drastically reduced genetic diversity and signature of selection
Source: BMC Plant Biol. 2014 Oct 19;14:279. doi: 10.1186/s12870-014-0279-2 (PMC4210547; doi:10.1186/s12870-014-0279-2)
Supplement: Additional file 6: — List of Arabidopsis candidate genes proteins (TAIR 10). Output of the TBLASTn on tomato genome sequence (v2.40). [file 12870_2014_279_MOESM6_ESM.doc]

| **Additional file 6: List of Arabidopsis candidate proteins (TAIR 10). TBLASTn on tomato genome sequence (v2.40) output** |
| --- |
|  |
| Query: AT5G19280.1 | Symbols: KAPP, RAG1 | KAPP (KINASE ASSOCIATED PROTEIN PHOSPHATASE); phosphoprotein phosphatase/ protein kinase binding / protein serine/threonine phosphatase | chr5:6488450-6493182 FORWARD |
| Sbjct: Solyc01g079720.2.1 genomic_reference:SL2.40ch01 gene_region:71336146-71351250 transcript_region:SL2.40ch01:71336146..71351250- go_terms:GO:0004721,GO:0019901 functional_description:"Kinase-associated protein phosphatase 1 (AHRD V1 **** A6N8J3_SOLPE); contains Interpro domain(s) IPR015655 Protein phosphatase 2C " |
| tblastn // Sbjct : 362 2023 2285 +2 // Query : 27 580 581 0 // S=623 E=1e-178 I=313/565 (55%) Pos=418/565 (73%) |
|  |
| Query: AT2G45190.1 | Symbols: AFO, FIL, YAB1 | AFO (ABNORMAL FLORAL ORGANS); protein binding / transcription factor/ transcription regulator | chr2:18628450-18630552 REVERSE |
| Sbjct: Solyc01g091010.2.1 genomic_reference:SL2.40ch01 gene_region:76475369-76478983 transcript_region:SL2.40ch01:76475369..76478983- go_terms:GO:0005515,GO:0003700 functional_description:"YABBY-like transcription factor CRABS CLAW-like protein (AHRD V1 **-* Q6SRZ7_ANTMA); contains Interpro domain(s) IPR006780 YABBY protein " |
| tblastn // Sbjct : 252 836 1077 +3 // Query : 22 229 229 0 // S=250 E=5e-67 I=137/210 (65%) Pos=160/210 (76%) |
|  |
| Query: AT2G34710.1 | Symbols: PHB, ATHB14, ATHB-14, PHB-1D | PHB (PHABULOSA); DNA binding / transcription factor | chr2:14639548-14643993 REVERSE |
| Sbjct: Solyc02g024070.2.1 genomic_reference:SL2.40ch02 gene_region:15627267-15633567 transcript_region:SL2.40ch02:15627267..15633567+ go_terms:GO:0005515,GO:0003677 functional_description:"Class III homeodomain-leucine zipper (AHRD V1 ***- Q1WD30_GINBI); contains Interpro domain(s) IPR013978 MEKHLA " |
| tblastn // Sbjct : 282 2783 2974 +3 // Query : 21 852 852 0 // S=1340 E=0.0 I=657/839 (78%) Pos=728/839 (86%) |
|  |
| Query: AT4G18960.1 | Symbols: AG | AG (AGAMOUS); DNA binding / transcription factor | chr4:10383917-10388272 FORWARD |
| Sbjct: Solyc02g071730.2.1 genomic_reference:SL2.40ch02 gene_region:35671866-35677320 transcript_region:SL2.40ch02:35671866..35677320- go_terms:GO:0005515,GO:0003700 functional_description:"MADS-box transcription factor AGAMOUS (AHRD V1 **-* Q8GTY3_HELAN); contains Interpro domain(s) IPR002100 Transcription factor, MADS-box IPR002487 Transcription factor, K-box " |
| tblastn // Sbjct : 126 863 993 +3 // Query : 3 252 252 0 // S=329 E=1e-90 I=170/254 (66%) Pos=197/254 (77%) |
|  |
| Query: AT2G17950.1 | Symbols: WUS, PGA6, WUS1 | WUS (WUSCHEL); DNA binding / protein binding / transcription factor/ transcription regulator | chr2:7809100-7810671 REVERSE |
| Sbjct: Solyc02g083950.2.1 genomic_reference:SL2.40ch02 gene_region:41768119-41769544 transcript_region:SL2.40ch02:41768119..41769544- go_terms:GO:0006355 functional_description:"WUSCHEL-related homeobox-containing protein 4 (AHRD V1 *-*- C0LAL8_9MAGN); contains Interpro domain(s) IPR001356 Homeobox " |
| tblastn // Sbjct : 92 307 1006 +2 // Query : 30 101 292 0 // S=132 E=2e-31 I=56/72 (77%) Pos=69/72 (95%) |
|  |
| Query: AT1G48410.1 | Symbols: AGO1 | AGO1 (ARGONAUTE 1); endoribonuclease/ miRNA binding / protein binding / siRNA binding | chr1:17886285-17891892 REVERSE |
| Sbjct: Solyc06g072300.2.1 genomic_reference:SL2.40ch06 gene_region:40960724-40969540 transcript_region:SL2.40ch06:40960724..40969540- go_terms:GO:0019899 functional_description:"ARGONAUTE 1 (AHRD V1 ***- D6RUV9_TOBAC); contains Interpro domain(s) IPR003165 Stem cell self-renewal protein Piwi " |
| tblastn // Sbjct : 2106 4931 5143 +3 // Query : 115 1048 1048 0 // S=1578 E=0.0 I=766/943 (81%) Pos=824/943 (87%) |
|  |
| Query: AT1G15750.1 | Symbols: WSIP1, TPL | TPL (TOPLESS); protein binding / protein homodimerization/ transcription repressor | chr1:5415086-5420359 REVERSE |
| Sbjct: Solyc03g117360.2.1 genomic_reference:SL2.40ch03 gene_region:60573693-60584026 transcript_region:SL2.40ch03:60573693..60584026+ go_terms:GO:0042803,GO:0016564 functional_description:"WD-40 repeat protein-like (Fragment) (AHRD V1 *--- Q1HIU4_9ROSI); contains Interpro domain(s) IPR017986 WD40 repeat, region " |
| tblastn // Sbjct : 1 3390 3774 +1 // Query : 1 1131 1131 0 // S=1865 E=0.0 I=898/1134 (79%) Pos=975/1134 (85%) |
|  |
| Query: AT1G52150.1 | Symbols: ATHB-15, ATHB15, CNA, ICU4 | ATHB-15; DNA binding / transcription factor | chr1:19409913-19413961 REVERSE |
| Sbjct: Solyc03g120910.2.1 genomic_reference:SL2.40ch03 gene_region:63195847-63202781 transcript_region:SL2.40ch03:63195847..63202781+ go_terms:GO:0003700 functional_description:"Class III homeodomain-leucine zipper (AHRD V1 ***- Q1WD30_GINBI); contains Interpro domain(s) IPR013978 MEKHLA " |
| tblastn // Sbjct : 308 2809 2982 +2 // Query : 4 836 836 0 // S=1466 E=0.0 I=707/838 (84%) Pos=764/838 (91%) |
|  |
| Query: AT1G55580.1 | Symbols: LAS, SCL18 | LAS (Lateral Suppressor); transcription factor | chr1:20764106-20765443 FORWARD |
| Sbjct: Solyc07g066250.1.1 evidence_code:10F0H1E0IEG genomic_reference:SL2.40ch07 gene_region:64958148-64959434 transcript_region:SL2.40ch07:64958148..64959434+ go_terms:GO:0003700 functional_description:"GRAS family transcription factor (Fragment) (AHRD V1 **-* B1Q3B1_BRACM); contains Interpro domain(s) IPR005202 GRAS transcription factor " |
| tblastn // Sbjct : 145 1284 1287 +1 // Query : 41 445 445 0 // S=382 E=1e-106 I=205/405 (50%) Pos=258/405 (63%) |
|  |
| Query: AT1G62360.1 | Symbols: STM, BUM1, SHL, WAM1, BUM, WAM | STM (SHOOT MERISTEMLESS); transcription factor | chr1:23058796-23061722 REVERSE |
| Sbjct: Solyc02g081120.2.1 genomic_reference:SL2.40ch02 gene_region:39767063-39773953 transcript_region:SL2.40ch02:39767063..39773953+ go_terms:GO:0005515 functional_description:"Knotted-1-like homeobox protein H1 (AHRD V1 ***- Q8GUS6_TOBAC); contains Interpro domain(s) IPR005541 KNOX2 " |
| tblastn // Sbjct : 646 1410 1613 +1 // Query : 126 381 382 0 // S=370 E=1e-103 I=195/258 (75%) Pos=206/258 (79%) |
|  |
| Query: AT1G65380.1 | Symbols: CLV2, AtRLP10 | CLV2 (clavata 2); protein binding / receptor signaling protein | chr1:24286943-24289105 FORWARD |
| Sbjct: Solyc04g056640.1.1 evidence_code:10F0H1E1IEG genomic_reference:SL2.40ch04 gene_region:53775339-53777579 transcript_region:SL2.40ch04:53775339..53777579+ go_terms:GO:0004675 functional_description:"LRR receptor-like serine/threonine-protein kinase, RLP" |
| tblastn // Sbjct : 163 2226 2241 +1 // Query : 33 719 720 0 // S=707 E=0.0 I=385/695 (55%) Pos=485/695 (69%) |
|  |
| Query: AT4G37750.1 | Symbols: ANT, DRG, CKC, CKC1 | ANT (AINTEGUMENTA); DNA binding / transcription factor | chr4:17739782-17742189 FORWARD |
| Sbjct: Solyc02g092050.2.1 genomic_reference:SL2.40ch02 gene_region:47836352-47839882 transcript_region:SL2.40ch02:47836352..47839882- go_terms:GO:0003677 functional_description:"AP2-like ethylene-responsive transcription factor At1g16060 (AHRD V1 *-*- AP2L1_ARATH); contains Interpro domain(s) IPR001471 Pathogenesis-related transcriptional factor and ERF, DNA-binding " |
| tblastn // Sbjct : 337 1737 2275 +1 // Query : 66 491 555 0 // S=421 E=1e-118 I=246/496 (49%) Pos=286/496 (57%) |
|  |
| Query: AT4G24190.1 | Symbols: SHD, HSP90.7 | SHD (SHEPHERD); ATP binding / unfolded protein binding | chr4:12551902-12555851 REVERSE |
| Sbjct: Solyc04g081570.2.1 genomic_reference:SL2.40ch04 gene_region:63110553-63116088 transcript_region:SL2.40ch04:63110553..63116088- go_terms:GO:0042623,GO:0050750 functional_description:"Chaperone protein htpG (AHRD V1 **-- HTPG_MYCA1); contains Interpro domain(s) IPR015566 Molecular chaperone, heat shock protein, endoplasmin " |
| tblastn // Sbjct : 1117 2421 2792 +1 // Query : 340 775 823 0 // S=773 E=0.0 I=372/436 (85%) Pos=410/436 (94%) |
|  |
| Query: AT1G75820.1 | Symbols: CLV1, FAS3, FLO5 | CLV1 (CLAVATA 1); ATP binding / kinase/ protein serine/threonine kinase/ receptor signaling protein serine/threonine kinase | chr1:28463631-28466652 REVERSE |
| Sbjct: Solyc04g081590.2.1 genomic_reference:SL2.40ch04 gene_region:63126375-63130644 transcript_region:SL2.40ch04:63126375..63130644- go_terms:GO:0004675 functional_description:"Receptor like kinase, RLK" |
| tblastn // Sbjct : 98 2965 3304 +2 // Query : 24 976 980 0 // S=1246 E=0.0 I=607/957 (63%) Pos=743/957 (77%) |
|  |
| Query: AT4G28190.1 | Symbols: ULT1, ULT | ULT1 (ULTRAPETALA1); DNA binding | chr4:13985753-13987050 FORWARD |
| Sbjct: Solyc07g054450.2.1 genomic_reference:SL2.40ch07 gene_region:60092916-60096795 transcript_region:SL2.40ch07:60092916..60096795+ go_terms:GO:0005634 functional_description:"Transcription factor (Fragment) (AHRD V1 ***- D6MKF6_9ASPA); contains Interpro domain(s) IPR000770 SAND " |
| tblastn // Sbjct : 209 874 1103 +2 // Query : 12 237 237 0 // S=336 E=7e-93 I=157/226 (69%) Pos=189/226 (83%) |
|  |
| Query: AT1G30490.1 | Symbols: PHV, ATHB9 | PHV (PHAVOLUTA); DNA binding / protein binding / transcription factor | chr1:10796328-10800744 REVERSE |
| Sbjct: Solyc02g024070.2.1 genomic_reference:SL2.40ch02 gene_region:15627267-15633567 transcript_region:SL2.40ch02:15627267..15633567+ go_terms:GO:0005515,GO:0003677 functional_description:"Class III homeodomain-leucine zipper (AHRD V1 ***- Q1WD30_GINBI); contains Interpro domain(s) IPR013978 MEKHLA " |
| tblastn // Sbjct : 282 2783 2974 +3 // Query : 17 841 841 0 // S=1304 E=0.0 I=640/841 (76%) Pos=719/841 (85%) |
|  |
| Query: AT5G43810.1 | Symbols: ZLL, PNH, AGO10 | ZLL (ZWILLE); translation initiation factor | chr5:17611939-17616562 FORWARD |
| Sbjct: Solyc09g082830.2.1 genomic_reference:SL2.40ch09 gene_region:63888767-63895211 transcript_region:SL2.40ch09:63888767..63895211- go_terms:GO:0019899 functional_description:"ARGONAUTE 1 (AHRD V1 ***- D6RUV9_TOBAC); contains Interpro domain(s) IPR003165 Stem cell self-renewal protein Piwi " |
| tblastn // Sbjct : 474 3050 3240 +3 // Query : 128 988 988 0 // S=1578 E=0.0 I=753/862 (87%) Pos=810/862 (93%) |
|  |
| Query: AT5G60690.1 | Symbols: REV, IFL, IFL1 | REV (REVOLUTA); DNA binding / lipid binding / transcription factor | chr5:24397734-24401933 FORWARD |
| Sbjct: Solyc11g069470.1.1 evidence_code:10F1H1E1IEG genomic_reference:SL2.40ch11 gene_region:51150759-51156271 transcript_region:SL2.40ch11:51150759..51156271+ go_terms:GO:0006355,GO:0045449 functional_description:"Class III homeodomain-leucine zipper (AHRD V1 ***- Q1WD30_GINBI); contains Interpro domain(s) IPR013978 MEKHLA " |
| tblastn // Sbjct : 1 2523 2526 +1 // Query : 1 842 842 0 // S=1377 E=0.0 I=659/845 (77%) Pos=729/845 (86%) |
|  |
| Query: AT5G49720.1 |
| Sbjct: Solyc01g102580.2.1 genomic_reference:SL2.40ch01 gene_region:83117822-83121772 transcript_region:SL2.40ch01:83117822..83121772- go_terms:GO:0008810 functional_description:"Endo-1 4-beta-glucanase (AHRD V1 ***- O04890_SOLLC); contains Interpro domain(s) IPR008928 Six-hairpin glycosidase-like IPR018221 Glycoside hydrolase, family 9, active site IPR001701 Glycoside hydrolase, family 9 " |
| tblastn // Sbjct : 323 2125 2515 +2 // Query : 1 605 621 0 // S=870 E=0.0 I=423/605 (69%) Pos=461/605 (76%) |
|  |
| Query: AT1G30950.1 |
| Sbjct: Solyc02g081670.1.1 evidence_code:10F1H1E1IEG genomic_reference:SL2.40ch02 gene_region:40120235-40121602 transcript_region:SL2.40ch02:40120235..40121602+ go_terms:GO:0008134,GO:0004842 functional_description:"Fimbriata (Fragment) (AHRD V1 **-- Q6QVW9_MIMLE); contains Interpro domain(s) IPR001810 Cyclin-like F-box " |
| tblastn // Sbjct : 127 1353 1368 +1 // Query : 41 438 442 0 // S=492 E=1e-139 I=243/410 (59%) Pos=298/410 (72%) |
|  |
| Query: AT5G66240.2 |
| Sbjct: Solyc02g083940.2.1 genomic_reference:SL2.40ch02 gene_region:41756601-41762285 transcript_region:SL2.40ch02:41756601..41762285+ go_terms:GO:0042800 functional_description:"WD repeat protein-like (AHRD V1 **-- Q8RXD8_ARATH); contains Interpro domain(s) IPR020472 G-protein beta WD-40 repeat, region " |
| tblastn // Sbjct : 16 996 1020 +1 // Query : 5 331 331 0 // S=544 E=1e-155 I=263/327 (80%) Pos=292/327 (89%) |
|  |
| Query: AT5G66750.1 |
| Sbjct: Solyc02g062780.2.1 genomic_reference:SL2.40ch02 gene_region:29133439-29143895 transcript_region:SL2.40ch02:29133439..29143895- go_terms:GO:0004003 functional_description:"Chromodomain-helicase-DNA- binding protein 6 (AHRD V1 **-* CHD6_HUMAN); contains Interpro domain(s) IPR000330 SNF2-related " |
| tblastn // Sbjct : 97 2313 2547 +1 // Query : 27 764 764 0 // S=1028 E=0.0 I=509/742 (68%) Pos=592/742 (79%) |
|  |
| Query: AT2G18500.1 |
| Sbjct: Solyc03g034100.2.1 genomic_reference:SL2.40ch03 gene_region:10070572-10072340 transcript_region:SL2.40ch03:10070572..10072340+ functional_description:"Plant-specific domain TIGR01568 family protein (AHRD V1 *--- B6U561_MAIZE); contains Interpro domain(s) IPR006458 Protein of unknown function DUF623, plant " |
| tblastn // Sbjct : 942 1190 1344 +3 // Query : 206 290 315 0 // S=99.0 E=4e-21 I=52/85 (61%) Pos=58/85 (68%) |
|  |
| Query: AT1G14870.1 |
| Sbjct: Solyc01g005470.2.1 genomic_reference:SL2.40ch01 gene_region:322628-324932 transcript_region:SL2.40ch01:322628..324932- functional_description:"Cell number regulator 10 (AHRD V1 **-- D9HP26_MAIZE); contains Interpro domain(s) IPR006461 Protein of unknown function Cys-rich " |
| tblastn // Sbjct : 163 573 750 +1 // Query : 13 152 152 0 // S=196 E=5e-51 I=94/140 (67%) Pos=109/140 (77%) |
|  |
| Query: AT5G03960.1 |
| Sbjct: Solyc04g016480.2.1 genomic_reference:SL2.40ch04 gene_region:7305326-7308804 transcript_region:SL2.40ch04:7305326..7308804- go_terms:GO:0005516 functional_description:"Calmodulin binding protein (AHRD V1 *--* B4FU94_MAIZE); contains Interpro domain(s) IPR000048 IQ calmodulin-binding region " |
| tblastn // Sbjct : 123 1211 1551 +3 // Query : 1 358 403 0 // S=107 E=2e-23 I=99/371 (26%) Pos=164/371 (44%) |
|  |
| Query: AT2G28290.1 |
| Sbjct: Solyc11g062010.1.1 evidence_code:10F0H1E0IEG genomic_reference:SL2.40ch11 gene_region:45838303-45866988 transcript_region:SL2.40ch11:45838303..45866988+ go_terms:GO:0005524 functional_description:"Chromodomain-helicase-DNA-binding protein 1 (AHRD V1 *--- CHD1_DROME); contains Interpro domain(s) IPR000330 SNF2-related " |
| tblastn // Sbjct : 1291 4803 8004 +1 // Query : 339 1485 3574 0 // S=1377 E=0.0 I=708/1191 (59%) Pos=828/1191 (69%) |
|  |
| Query: AT5G65700.1 |
| Sbjct: Solyc02g091840.2.1 genomic_reference:SL2.40ch02 gene_region:47648877-47652967 transcript_region:SL2.40ch02:47648877..47652967+ go_terms:GO:0004675 functional_description:"Receptor like kinase, RLK" |
| tblastn // Sbjct : 265 3222 3516 +1 // Query : 12 999 1003 0 // S=1538 E=0.0 I=751/991 (75%) Pos=851/991 (85%) |
|  |
| Query: AT5G03840.1 |
| Sbjct: Solyc06g074350.2.1 genomic_reference:SL2.40ch06 gene_region:42361623-42363883 transcript_region:SL2.40ch06:42361623..42363883+ go_terms:GO:0005515,GO:0008429 functional_description:"self-pruning" |
| tblastn // Sbjct : 82 606 859 +1 // Query : 4 177 177 0 // S=264 E=2e-71 I=121/175 (69%) Pos=151/175 (86%) |
|  |
| Query: AT3G55510.1 |
| Sbjct: Solyc05g023710.2.1 genomic_reference:SL2.40ch05 gene_region:29024158-29035186 transcript_region:SL2.40ch05:29024158..29035186+ functional_description:"Nucleolar complex protein 2 homolog (AHRD V1 ***- C0H9R3_SALSA); contains Interpro domain(s) IPR005343 Uncharacterised protein family UPF0120 " |
| tblastn // Sbjct : 349 1800 2188 +1 // Query : 107 593 594 0 // S=461 E=1e-130 I=241/493 (48%) Pos=337/493 (68%) |
