# Supplementary material for: Genes involved in floral meristem in tomato exhibit drastically reduced genetic diversity and signature of selection
Source: BMC Plant Biol. 2014 Oct 19;14:279. doi: 10.1186/s12870-014-0279-2 (PMC4210547; doi:10.1186/s12870-014-0279-2)
Supplement: Additional file 7: — Information on the 96 accessions used in the association study and diversity analysis: List of accessions, subgroups and phenotypic data. Species are indicated as well as values for fruits weigh (FW), locule number (LCN) and fruit shape index (FSI). [file 12870_2014_279_MOESM7_ESM.doc]

| **Additional file 7: Phenotypic data Information on 96 accessions used in the association study and diversity analysis** | | | | |  |  |
| --- | --- | --- | --- | --- | --- | --- |
| Accession Number | Accession Name | species | mature fruit color | Traits ^a^ | |  |
|  |  |  |  | FW (g) | LCN | FSI |
| CR001 | Cervil | *S.l.cerasiforme* | red | 5.796018 | 2.183998 | 0.8752 |
| CR002 | Levovil | *S.lycopersicum* | red | 109.131417 | 3.910619 | 0.7959 |
| CR003 | Ferum | *S.lycopersicum* | red | 109.664431 | 2.23837 | 0.8806 |
| CR004 | M-82 | *S.lycopersicum* | red | 62.601018 | 2.452516 | 1.0552 |
| CR014 | Clémentine | *S.l.cerasiforme* | red | 5.404351 | 2.252516 | 0.9989 |
| CR020 | San Marzano | *S.lycopersicum* | red | 70.004351 | 2.245109 | 1.2742 |
| CR028 | Plovdiv XXIVa | *S.l.cerasiforme* | red | 41.579351 | 2.050665 | 1.094 |
| CR031 | Microtom | *S.lycopersicum* | red | 6.914351 | 3.167331 | 0.8761 |
| CR032 | Moneymaker | *S.lycopersicum* | red | 99.536018 | 2.491934 | 0.8409 |
| CR056 | Wva 700 | *S.l.cerasiforme* | red | 4.254351 | 2.100665 | 0.9684 |
| CR058 | Wva 106 | *S.l.cerasiforme* | red | 9.687685 | 2.717331 | 0.8483 |
| CR062 | LA 1478 | *S.pimpinellifolium* | red | 2.054351 | 2.300665 | 0.9736 |
| CR068 | N° 108 Red Currant | *S.pimpinellifolium* | red | 2.004351 | 1.983998 | 1.0114 |
| CR070 | N° 2909 Lycopersicon sp. | *S.l.cerasiforme* | red | 5.121018 | 2.050665 | 1.1414 |
| CR072 | N° 2921 Lyc. Pimpinellifolium | *S.pimpinellifolium* | red | 2.154351 | 2.017331 | 0.9724 |
| CR075 | N° 4156 Blumen Strauss | *S.pimpinellifolium* | red | 1.662685 | 1.933998 | 1.2388 |
| CR076 | N° 135 Green Gage | *S.l.cerasiforme* | red | 39.904351 | 2.133998 | 0.8765 |
| CR077 | N°1565 | *S.l.cerasiforme* | red | 10.187685 | 2.283998 | 0.8364 |
| CR078 | N° 2759 Enano | *S.l.cerasiforme* | red | 34.837685 | 3.333998 | 0.8565 |
| CR079 | N° 933 | *S.l.cerasiforme* | red | 33.637685 | 3.817331 | 0.8292 |
| CR093 | N° 2257 Dikorastushii... | *S.l.cerasiforme* | red | 23.546018 | 3.583998 | 0.7908 |
| CR094 | N° 1011 Srednei Velichiny | *S.lycopersicum* | red | 23.297685 | 2.978442 | 0.9184 |
| CR097 | N° 347 Yablochnyi | *S.l.cerasiforme* | red | 30.487685 | 2.350665 | 0.8131 |
| CR098 | N° 795 Pescio | *S.l.cerasiforme* | red | 22.107685 | 3.817331 | 0.8917 |
| CR101 | N° 884 Alagabotskii | *S.l.cerasiforme* | red | 24.487685 | 3.350665 | 0.7787 |
| CR102 | N° 739 | *S.l.cerasiforme* | red | 54.129351 | 7.521498 | 0.6122 |
| CR106 | LA 1025 | *S.l.cerasiforme* | red | 15.079351 | 2.100665 | 0.9139 |
| CR108 | LA 1231 | *S.l.cerasiforme* | red | 4.987685 | 2.183998 | 0.9525 |
| CR110 | LA 1307 | *S.l.cerasiforme* | red | 14.746018 | 2.152516 | 0.9598 |
| CR117 | LA 1388 | *S.l.cerasiforme* | red | 16.887685 | 4.046961 | 0.7936 |
| CR118 | LA 1420 | *S.l.cerasiforme* | red | 39.682685 | 5.132146 | 0.8141 |
| CR122 | LA 1456 | *S.l.cerasiforme* | red | 4.964351 | 2.000665 | 1.0645 |
| CR123 | LA 1461 | *S.l.cerasiforme* | red | 3.934351 | 2.017331 | 0.9056 |
| CR124 | LA 1464 | *S.l.cerasiforme* | red | 3.249351 | 2.017331 | 0.9402 |
| CR125 | LA 1482 | *S.l.cerasiforme* | red | 9.659351 | 2.300665 | 0.9018 |
| CR129 | LA 0147 | *S.lycopersicum* | red | 116.771018 | 3.939553 | 0.7893 |
| CR130 | LA 0172 | *S.l.cerasiforme* | red | 37.681018 | 3.483998 | 0.76 |
| CR133 | LA 0409 | *S.lycopersicum* | red | 116.722685 | 15.479236 | 0.7208 |
| CR134 | LA 0466 | *S.lycopersicum* | red | 208.894351 | 12.711776 | 0.7041 |
| CR136 | LA 0473 | *S.lycopersicum* | red | 49.887685 | 9.533998 | 0.6327 |
| CR145 | LA 1543 | *S.l.cerasiforme* | red | 11.267685 | 2.150665 | 1.3821 |
| CR149 | LA 2095 | *S.l.cerasiforme* | red | 26.904351 | 3.598813 | N/A |
| CR150 | LA 2131 | *S.l.cerasiforme* | red | 40.357685 | 4.511776 | 0.8518 |
| CR152 | LA 2307 | *S.l.cerasiforme* | red | 25.999351 | 3.328442 | 0.7592 |
| CR153 | LA 2308 | *S.l.cerasiforme* | red | 27.699351 | 2.915479 | 0.8699 |
| CR155 | LA 2402 | *S.l.cerasiforme* | red | 6.771018 | 2.233998 | 0.7049 |
| CR156 | LA 2619 | *S.l.cerasiforme* | red | 13.771018 | 4.133998 | 0.7519 |
| CR158 | LA 2675 | *S.l.cerasiforme* | red | 4.987685 | 2.000665 | 0.9392 |
| CR159 | LA 2688 | *S.l.cerasiforme* | red | 4.337685 | 2.000665 | 0.9958 |
| CR163 | LA 0400 | *S.pimpinellifolium* | red | 2.104351 | 2.083998 | 0.9062 |
| CR164 | LA 0411 | *S.pimpinellifolium* | red | 3.137685 | 2.150665 | 0.9176 |
| CR169 | LA 1371 | *S.pimpinellifolium* | red | 2.304351 | 2.033998 | 0.9639 |
| CR173 | LA 1547 | *S.pimpinellifolium* | red | 3.421018 | 2.000665 | 0.9174 |
| CR186 | LA 1689 | *S.pimpinellifolium* | red | 2.204351 | 2.133998 | 0.874 |
| CR199 | tomate Richter's | *S.l.cerasiforme* | red | 3.821018 | 2.067331 | 0.9472 |
| CR202 | CGN 18399 | *S.l.cerasiforme* | red | 6.454351 | 2.083998 | 0.91 |
| CR203 | LA 1589 | *S.pimpinellifolium* | red | 2.404351 | 2.083998 | 0.8996 |
| CR205 | L. pimpinellifolium atypique, site 10 (F300045) | *S.l.cerasiforme* | red | 10.421018 | 2.150665 | 1.1253 |
| CR234 | Atom | *S.l.cerasiforme* | red | 26.496018 | 2.533998 | 0.9738 |
| CR236 | PI 365923 | *S.l.cerasiforme* | red | 15.321018 | 2.083998 | 0.8764 |
| CR238 | PI 129088 | *S.l.cerasiforme* | red | 12.334351 | 3.154831 | 0.9843 |
| CR240 | L 285 | *S.l.cerasiforme* | red | 15.962685 | 2.158998 | 0.9402 |
| CR244 | Yellow Pear | *S.l.cerasiforme* | red | 19.054351 | 2.350665 | 1.5405 |
| CR249 | Cherry Gold | *S.l.cerasiforme* | red | 7.526018 | 2.55622 | 0.884 |
| CR250 | Cherry VFNT | *S.l.cerasiforme* | red | 21.601018 | 2.000665 | 0.8671 |
| CR252 | Droplet | *S.l.cerasiforme* | red | 16.712685 | 2.217331 | 1.4354 |
| CR253 | Monplaisir | *S.l.cerasiforme* | red | 22.487685 | 2.217331 | 0.8812 |
| CR254 | Farthest North | *S.l.cerasiforme* | red | 8.912685 | 3.133998 | 0.9394 |
| CR256 | Minibel | *S.l.cerasiforme* | red | 19.454351 | 4.167331 | 0.7867 |
| CR258 | Ohmiya Suncherry | *S.l.cerasiforme* | red | 13.821018 | 2.100665 | 0.8732 |
| CR267 | Tiny tim | *S.l.cerasiforme* | red | 11.079351 | 2.800665 | 0.8867 |
| CR271 | Celsior | *S.l.cerasiforme* | red | 12.121018 | 2.017331 | 1.6728 |
| CR273 | Orange Cocktail | *S.lycopersicum* | red | 60.707685 | 4.072093 | 0.9093 |
| CR274 | Marpha n°2 | *S.l.cerasiforme* | red | 8.537685 | 3.300665 | 0.7901 |
| CR275 | Cerise Ildi | *S.l.cerasiforme* | red | 7.654351 | 2.600665 | 1.1476 |
| CR279 | Cerise Orange d'Uzès | *S.l.cerasiforme* | red | 13.821018 | 2.267331 | 0.9619 |
| CR280 | Cerise du sud ouest n° 2 | *S.l.cerasiforme* | red | 10.221018 | 2.150665 | 0.8816 |
| CR284 | cerise rose | *S.l.cerasiforme* | red | 10.454351 | 2.800665 | 0.9276 |
| CR287 | Cisterno | *S.l.cerasiforme* | red | 21.554351 | 2.350665 | 0.8742 |
| CR288 | Criollo | *S.l.cerasiforme* | red | 26.109351 | 3.672887 | 0.7505 |
| CR291 | Pyriforme | *S.l.cerasiforme* | red | 10.037685 | 2.033998 | 1.3245 |
| CR292 | 8 bis | *S.l.cerasiforme* | red | 20.654351 | 2.217331 | 0.9473 |
| CR293 | Costa Rica | *S.l.cerasiforme* | red | 15.871018 | 3.167331 | 0.8772 |
| CR294 | Phyra | *S.l.cerasiforme* | red | 5.254351 | 2.217331 | 1.1248 |
| CR296 | Poire jaune | *S.l.cerasiforme* | red | 16.942685 | 2.229831 | 1.5153 |
| CR317 | Heinz 1706 | *S.lycopersicum* | red | 43.704351 | 2.500665 | 1.3732 |
| CR321 | Edkawy | *S.lycopersicum* | red | 224.301018 | 11.182411 | 0.6418 |
| CR341 | Cra 66 | *S.lycopersicum* | red | 40.454351 | 5.183998 | 0.7926 |
| CR354 | Stupicke Polni Rane | *S.lycopersicum* | red | 61.211018 | 4.15622 | 0.8398 |
| CR359 | Muchamiel | *S.lycopersicum* | red | 172.942685 | 5.539553 | 0.8088 |
| CR206 | PI247087 | *S.habrochaites* | green | N/A | N/A | N/A |
| CR207 | LA716 | *S.pennellii* | green | N/A | N/A | N/A |
| CR228 | LA1401 | *S.chesmaniae* | orange | N/A | N/A | N/A |
| CR00X | X | *S.chmielewskii* | green | N/A | N/A | N/A |
| ^a^ Values for fruit weight (FW), locule number (LCN) are adjusted mean from two years of experiment | | | |  |  |  |
