# Supplementary material for: Genes involved in floral meristem in tomato exhibit drastically reduced genetic diversity and signature of selection
Source: BMC Plant Biol. 2014 Oct 19;14:279. doi: 10.1186/s12870-014-0279-2 (PMC4210547; doi:10.1186/s12870-014-0279-2)
Supplement: Additional file 8: — Primer list for re-sequenced candidates (v.2.40). [file 12870_2014_279_MOESM8_ESM.doc]

| **Additional file 8:** Primer list for re-sequenced candidates (v.2.40) | | |
| --- | --- | --- |
| Fragment_ID | Primer Seq F | Primer R Seq |
| CLV1 | ACAACAGGGCAAAGACAAATCAA | CAGCTTCCAATTCACCACAAAAC |
| CNA | TGGCTGCTAAACTCCTTCTACCC | TGTTGGCTGCTTCAACTTTCTTC |
| FIL-YAB1 | GGGATCAGAGTCAAAACCCATCT | CAAACGTCCCTTTCTCACTCCTT |
| KAPP | GATTTCATATCGCACGACCAAAC | AGTCCCAGGCAAACCAAAATAGA |
| LAS | CTTTTCTACATTGCCTCCAACCC | TTGTAATTTCCCACTCAAGCCAA |
| PHB | GGTGACACAGGGAAGGATTTAGG | TGGGGTTGGGAGAGTATTGTTTT |
| PHV | CACCAGAGTCCGATTTCCTTCTT | TTTATTCTCTCGCCTCTCGCTCT |
| SHD | ATTTGTGTAAATCAGCCAGCCAC | GAAGAACCAATTAAAATCCGCCA |
| STM | ATTCACACCTTTGTTGTGCGTCT | CCTTACCTCCCCAAATAGGCTTC |
| SYD | TAACTGCATCCTGCCCTACAAAA | TGTGCATCACCTACTTCACCAGA |
| TPL | TGTCGTGCACATTCATCTATCATCG | TCTTGGTCCTTCATCTATTCCAGGTA |
| ULT1 | ACGAGTCCATTCACCTTTTGCTT | TACCCCTCCACCTACTTGCCTAC |
| WUS | GATTTGGACTTTTGGGTTTGTCC | TCAATTTCAGTCCTTCTCTCCCA |
| ZLL-PNH-AGO10 | TTCCACTCCTATCAATGCTGCTC | AAAAACCCCAAGACCACTCAAAA |
| LC | CCGGTTCTTCTGAGCTTTCATTT | CGTGCCTTGAACATTTCTGTTTT |
| AGO1 | TGCTGTGTCAACTAGTTGCTTGTTCAGT | CAGGCTATGAAGAACACCGGAATGCTC |
| CLV2 | CGATAAGCGCAACATGACAAATGATTCCGA | TGTACTCAGTCTTACTCGCAGCGTTGA |
| *CONTROL* | CGTATACAGTTCACCTCTCTCCCACTGTA | GCTTCCCTTCATTAATTATACACCACTCTCTAT |
| REV | GGTACCTCTCCGTCTTAATTTACGTAACA | ACAATCATCTAAACAAGGTGTGACCATGC |
| FW2.2/ATPCR2 | GTGGCCAAGATCCAACCATTTCATCAATTG | GTTATCTTCTTCAACTCAACTGCTCCTTGTTCG |
| OVATE/ATOFP7 | CTCAGAAAGAGTATTGCAGGTTACTCTG | GACTTATGAGTTGTATTGGCCTGACG |
| KOR1 | GACGCCATAACTTCAACGAGTTCTTA | AACATGATCTCTATGATATGCATTGGC |
| RBL | CCACATAGGCTTATTGATAGAATCATTGC | GCATCTAATATTGGGAGATGCAACATG |
| ANT | GTGCCACATCAACTCATCAAGACATGA | CAGTATGCTGAACAGCATGCTATTGAC |
| SUN | CTTAGGATTCGTGCTATTGCTAGGTA | CTTCTGCAACACTATCGTGCGA |
| BAM1 | CATGGCCATACCAACACTACTAGGTC | GACTATCACTTCATGAGGTGCTTCAATGA |
| TD380 /DDM1 | TGCTTCTTCCCTGTTTCTTCTTTCT | AAGGTGACAACAAAACTAGCCTTCC |
| UFO | ATACCAAACCAAACGGAGGATAAGA | GCCTTCGGGAGTAAGATAACTGAAA |
| SP/TFL1 | AAGCTCCTTGTATGGGAAAAGATTG | GTTGTCGTACAGTTGATTAGACGGG |
| AG | GTGATCTAACCAGAGAGATCTCACCA | CTGGCATCAAGTTCATCTGCTGA |
